# Alcohol Intake and Risk of Hypertension: A Systematic Review and Dose-Response Meta-Analysis of Nonexperimental Cohort Studies

**DOI:** 10.1161/HYPERTENSIONAHA.124.22703

**Published:** 2024-06-12

**Authors:** Marta Cecchini, Tommaso Filippini, Paul K. Whelton, Inga Iamandii, Silvia Di Federico, Giuseppe Boriani, Marco Vinceti

**Affiliations:** 1 CREAGEN - Environmental, Genetic and Nutritional Epidemiology Research Center, Section of Public Health, Department of Biomedical, Metabolic and Neural Sciences (M.C., T.F., I.I., S.D.F., M.V.), University of Modena and Reggio Emilia, Modena, Italy.; 2 Unit of Cardiology, Department of Biomedical, Metabolic and Neural Sciences (G.B.), University of Modena and Reggio Emilia, Modena, Italy.; 3 School of Public Health, University of California Berkeley, Berkeley, CA (T.F.).; 4 Department of Epidemiology, Tulane University School of Public Health and Tropical Medicine, New Orleans, LA (P.K.W.).; 5 Department of Epidemiology, Boston University School of Public Health, Boston, MA (M.V.).

**Keywords:** alcohol intake, cardiovascular disease, hypertension, prevention, public health

## Abstract

**BACKGROUND:**

Alcohol consumption has been associated with higher blood pressure and an increased risk of hypertension. However, the possible exposure thresholds and effect-modifiers are uncertain.

**METHODS:**

We assessed the dose-response relationship between usual alcohol intake and hypertension incidence in nonexperimental cohort studies. After performing a systematic literature search through February 20, 2024, we retrieved 23 eligible studies. We computed risk ratios and 95% CI of hypertension incidence using a nonlinear meta-analytic model based on restricted cubic splines, to assess the dose-response association with alcohol consumption.

**RESULTS:**

We observed a positive and almost linear association between alcohol intake and hypertension risk with risk ratios of 0.89 (0.84–0.94), 1.11 (1.07–1.15), 1.22 (1.14–1.30), and 1.33 (1.18–1.49) for 0, 24, 36 and 48 g/d, respectively, using 12 g alcohol/d as the reference value. In sex-specific analyses, the association was almost linear in men over the entire range of exposure but only observed above 12 g/d in women, although with a steeper association at high levels of consumption compared with men. The increased risk of hypertension above 12 to 24 g alcohol/d was similar in Western and Asian populations and considerably greater in White than in Black populations, mainly due to the positive association in women at moderate-to-high intake.

**CONCLUSIONS:**

Overall, our results lend support to a causal association between alcohol consumption and risk of hypertension, especially above an alcohol intake of 12 g/d, and are consistent with recommendations to avoid or limit alcohol intake. Sex and ethnicity appear to be major effect-modifiers of such association.

Alcohol is a water-soluble, psychoactive, and addictive substance whose consumption may result in severe adverse health effects and about 3 million deaths each year globally, particularly at moderate-to-high consumption levels.^1,2^ To minimize these harmful effects, many governments and international agencies have implemented policies aimed at reducing alcohol intake.^3,4^

Alcohol consumption has been associated with a variety of cardiovascular disease outcomes, including cardiomyopathies, coronary artery disease, stroke, and increased blood pressure (BP),^5^ the latter end point of BP having been recently reviewed through a dose-response meta-analysis.^6^ However, uncertainties exist regarding its association with the risk of hard outcomes including hypertension, particularly at low levels of alcohol intake and whether sex and race modify the association.^7–9^ We took advantage of a new statistical technique that allows pooling and flexible modeling of the dose-response relationship between exposures and end points to assess the overall association between chronic alcohol intake and risk of hypertension in nonexperimental longitudinal studies.

## METHODS

This review was performed according to the Preferred Reporting Items for Systematic Reviews and Meta-Analyses guidelines,^10^ and its protocol was registered in PROSPERO (no. CRD42022314389).

### Literature Search and Study Selection

We conducted a systematic literature search in PubMed and Embase, using the keywords: “”alcohol’’, “hypertension,” “blood pressure,” “stroke,” “humans,” “cohort,” “case-cohort” and ‘’English’’ or “Italian,” for nonexperimental cohort studies published before February 20, 2024. Full literature search strategies are reported in Table S1. Using the Population, Exposure, Comparator, Outcome and Study Design approach,^11^ we included studies that reported the association between alcohol exposure and the incidence of hypertension. Details of study identification and selection are reported in the Supplemental Methods.

### Data Extraction

Three authors (M.C., I.I., and S.D.F.) extracted the information using a standardized data collection form. We recorded the following data: (1) study details (first author name, study design, publication year), (2) study participant characteristics (country, study cohort, sex, age, sample size, ethnicity, smoking assessment), (3) exposure characteristics (definition, data collection methods and categories of intake), (4) outcome characteristics: definition, outcome assessment methods, number of cases, hypertension risk ratios (RRs) with 95% CIs or SEs, duration of follow-up; (5) covariates employed in multivariable analyses. The risk estimates were recorded both for the overall study population and for different subgroups, as available. We systematically contacted the authors of eligible studies when information was missing but needed for inclusion of the study in our meta-analysis.

For the definition of hypertension incidence, we used the method employed in each study, for example, the use of BP thresholds or antihypertensive drug treatment for hypertension, and additionally evaluated the risk of bias assessment.

We extracted all details regarding alcohol consumption reported in each original publication and we used the methods for the assessment of alcohol dose in our analyses. For each intake category, we recorded the range of the dose or its mean or median value, depending on the available data. For studies reporting alcohol intake as frequency in drinks/wk or drinks/mo, we transformed the data into drinks/day by either dividing by 7 or dividing monthly intake reports by 30.4. We then converted the exposure to grams/day according to the size of a standard drink specifically considered in each study. When no information about the size of a standard drink was available, we considered that a World Health Organization country-specific standard drink contains 15 to 17.7 mL of alcohol corresponding to 12 to 14 g of alcohol.^2,12^ For 1 study carried out in China,^13^ the UK standard drink specification of 8 g of alcohol was used for the conversion.

### Risk of Bias Assessment

Details of the risk of bias assessment are reported in the Supplemental Methods. The internal validity of eligible studies was assessed using the Risk of Bias in Nonrandomized Studies of Exposure tool^14^ considering the following risk of bias domains: (1) confounding, (2) selection of participants into the study, (3) exposure assessment (4) departure from intended exposure, (5) missing data, (6) outcome ascertainment, and (7) selective reporting (Table S2). The overall results were tiered as follows: if at least 1 domain was found to identify a high risk of bias, the overall risk was considered high; if more than 1 domain was found to identify a moderate risk of bias, the overall risk was considered to be moderate; and if all domains were at low risk of bias, the overall risk was considered to be low.

### Data Analysis

To perform the dose-response meta-analysis, we used the 1-stage dose meta-analysis methodology, which allows modeling of RR across the range of alcohol exposure including studies with missing covariates (eg, number of incident cases and total study population) and provides a good approximation of the overall risk estimates using aggregated instead of the original data.^15^ The analysis was based on a restricted maximum likelihood random effects model and used restricted cubic splines with 3 knots at fixed percentiles (10th, 50th, and 90th) of alcohol intake distribution.^15^ We selected the optimal number of knots according to Akaike’s information criterion, and we used the knot placement recommended by Harrell.^16^ For each alcohol category, we abstracted the mean intake (or median in case of unavailability of means) from the study, together with the RR and its 95% lower and upper bounds, and the number of cases and person-years. When means or medians were not available, we assigned the midpoint in each of the exposure categories in the model. For open categories where the lower of the upper 95% bound was missing, we entered a value that was 20% higher or lower than the closest cut point.^17–19^ In addition to the overall analysis, we carried out subgroup analyses by stratifying for sex, geographic region, and ethnicity, whenever possible. We conducted sensitivity analyses that excluded studies that were categorized as being at high risk of bias, those using different cutoffs for definition of hypertension or not reporting such criteria, and studies in which smoking was not included in the multivariate model. We also assessed the influence of duration of follow-up by performing a stratified analysis using 20 years as the reference. We eventually provided a graphical overlay of study-specific trends using predicted study-specific curves showing the influence of variation across studies.^15,20,21^ All analyses were carried out by using Stata-MP software (v18.0, Stata Corp, College Station, TX, 2023), specifically the “meta,” “mkspline,” and “drmeta” routines.

## RESULTS

**Figure 1.**
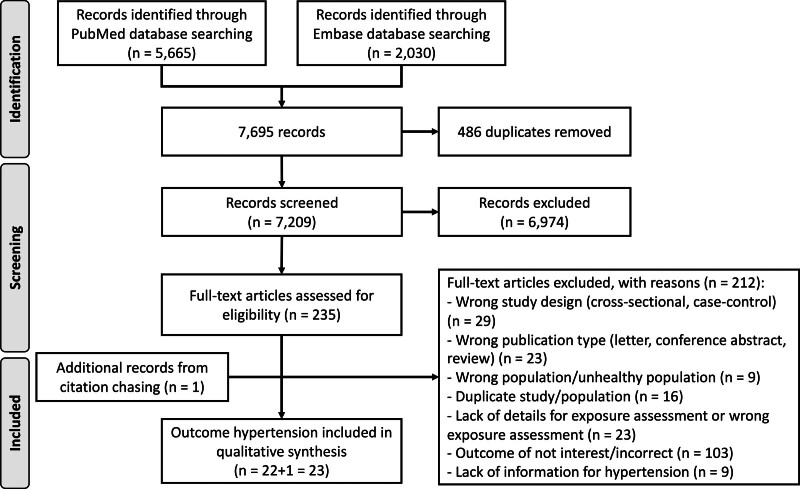
Flow chart of systematic literature search on alcohol exposure and hypertension up to February 20, 2024.

Details of the literature search are presented in the Preferred Reporting Items for Systematic Reviews and Meta-Analyses flowchart (Figure 1). We identified 7695 records of potential interest, from which 486 were removed as duplicates and 6974 were excluded after title and abstract screening, leaving 235 studies for full-text assessment. After evaluating the full text, we excluded 212 additional articles for the reasons reported in Figure 1. This included studies that had employed a cross-sectional or case-control design (n=29); letters, conference abstracts, and reviews (n=23); use of an ineligible study population (such as diseased participants; n=9); duplicate use of study populations (n=16); lack of exposure assessment information or inapplicable exposure assessment (n=23), incorrect (n=103) or unreported outcomes (n=9). One article was included following citation chasing.^22^

Details of the 23 articles included in our meta-analysis are presented in the Table.^9,13,22–43^ Eight studies were conducted in the United States,^24–26,31,38–40,42^ 1 in the United Kingdom,^27^ 4 in Japan,^32,33,35,36^ 5 in China,^13,23,28,37,41^ 3 in South Korea,^9,30,43^ and 2 in continental Europe (Spain and Eastern Finland).^22,34^ The studies were published between 1990 and 2023, with a sample size that exceeded 600 000 participants and 45 000 incident cases of hypertension during a median/mean follow-up period ranging from 2 to 22 years. Most of the studies included men and women, but 3 did not specify participant sex.^25,34,41^ Two studies were carried out in the same cohort^13,23^ but with different lengths of follow-up. The study with longer follow-up that only reported results for the overall population was included in the main analysis,^13^ while the other study with a shorter period of follow-up that reported results separately for men and women was considered for our sex-specific analyses.^23^ Participant ages ranged from 18 to 90 years. Most of the studies defined hypertension as an average systolic BP ≥140 mm Hg or diastolic BP ≥90 mm Hg, or treatment with antihypertensive medication. One study used higher cut points for diagnosis of hypertension (systolic BP ≥160 mm Hg or diastolic BP ≥95 mm Hg),^25^ another used a cut-point of ≥159/90 mm Hg until 1999, ≥140/90 mm Hg from 2000 to 2007, ≥130/85 mm Hg from 2008 to 2010, and subsequently ≥140/90 mm Hg or initiation of antihypertensive treatment,^36^ and 1 study used self-reported diagnosis of hypertension.^31^ All of the studies recorded incidence of hypertension as the primary outcome and diagnosis of hypertension was generally based on a review of medical records. In all studies, alcohol exposure was assessed using an adjusted dose value. Alcohol intake was assessed by dietary intake using simple or standardized questionnaires,^24,27,28,30–33,35,37–41^ interview-based questionnaire,^9,26,36^ or interviews by trained and certified health professionals.^13,22,23^ Four of the self-report studies used a food frequency questionnaire.^25,29,34,42^ Details of the methods used for exposure assessment and calculation of alcohol dose are presented in the Table. Specifically, 14 studies reported alcohol consumption using gram per day (or gram per week),^9,22,23,26,30–33,35–37,40–42^ and 2 other studies used unit per day^13^ and milliliters per day^29^ with direct conversion into gram per day. Conversely, 8 studies used frequency of drinks,^24,25,27,28,31,34,38,39^ with the consequent need to evaluate the amount of alcohol in the standard drink alternatively considered in each study to convert data for the analysis. All but 2 studies accounted for body mass index or waist circumference as potential confounders in the analysis.^28,31^ Most but not all^13,25,26,28,31,38,42^ studies included smoking as a possible confounding factor. All but 1 study adjusted for age.^31^ Additional confounders considered when available were family history of hypertension^24,27,30,40^ and cholesterol levels,^22,24,30,33,34,36,39–41^ potassium (3 studies) and sodium intake (4 studies),^22,29,34,37^ and diabetes (6 studies).^24,26,36,37,39,41^

**Table.**
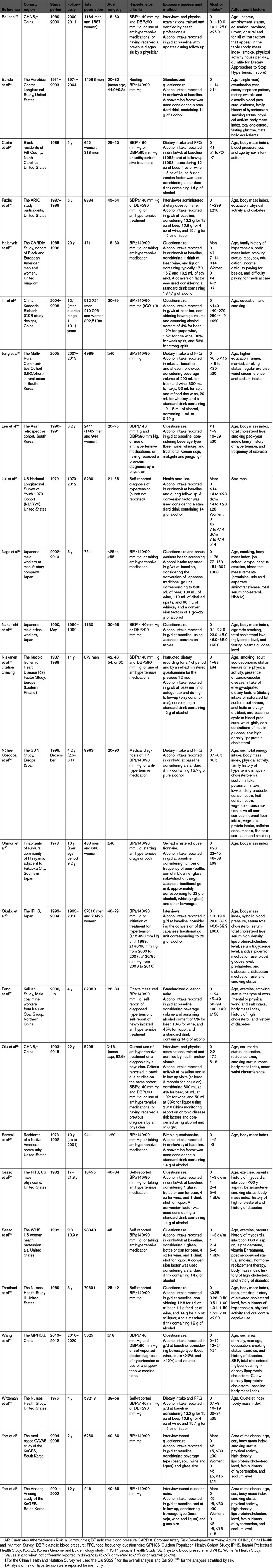
Characteristics of the Studies Included

Almost all of the studies assessed alcohol consumption at baseline only, with only a few evaluating drinking habits at follow-up visits^9,13,22,23,25,29,31^ and using the follow-up assessments for their estimation of hypertension risk. One study^25^ assessed how change in drinking habits affected hypertension risk through categorization into discontinued, continued, or newly initiated alcohol consumption. Another study^22^ reported risk of hypertension for alcohol consumption at follow-up using linear continuous increases only, hampering the use of their data in our analysis. One study^23^ only reported the risk of hypertension for baseline intake of alcohol despite assessing consumption at multiple time points. Conversely, 1 study^29^ reported alcohol consumption at baseline and follow-up and reported risk estimates for both assessments and their average. Finally, 2 studies assessed alcohol consumption for the overall period of investigation (baseline and follow-up), with 1 study evaluating multiple time points and estimating lifetime drinking patterns,^31^ and another study^9^ evaluating both baseline and follow-up and reporting a 10-year drinking habit.

Details of the risk of bias assessment and the overall risk of bias for the studies included in the final analysis are reported in Table S3. Only 3 of the studies were at high risk of bias.^22,30,31^ Five of the studies were judged to be at low risk of bias,^9,29,33,36,39^ while the remaining studies were at moderate risk of bias due to either confounding,^25,35,38,42^ exposure misclassification,^13,23,24,32,35,37,38,41^ missing data,^26,30,34,40,42^ and inadequate outcome ascertainment.^13,23^

Study-specific and summary RRs for hypertension incidence by comparing the highest with the lowest category of alcohol intake are reported in the forest plot in Figure S1. Overall, the RR of hypertension was 1.39 (95% CI, 1.25–1.56). The association was stronger in men than in women with RRs of 1.52 (95% CI, 1.35–1.71) and 1.18 (95% CI, 0.96–1.46), respectively (Figures S2 and S3).

**Figure 2.**
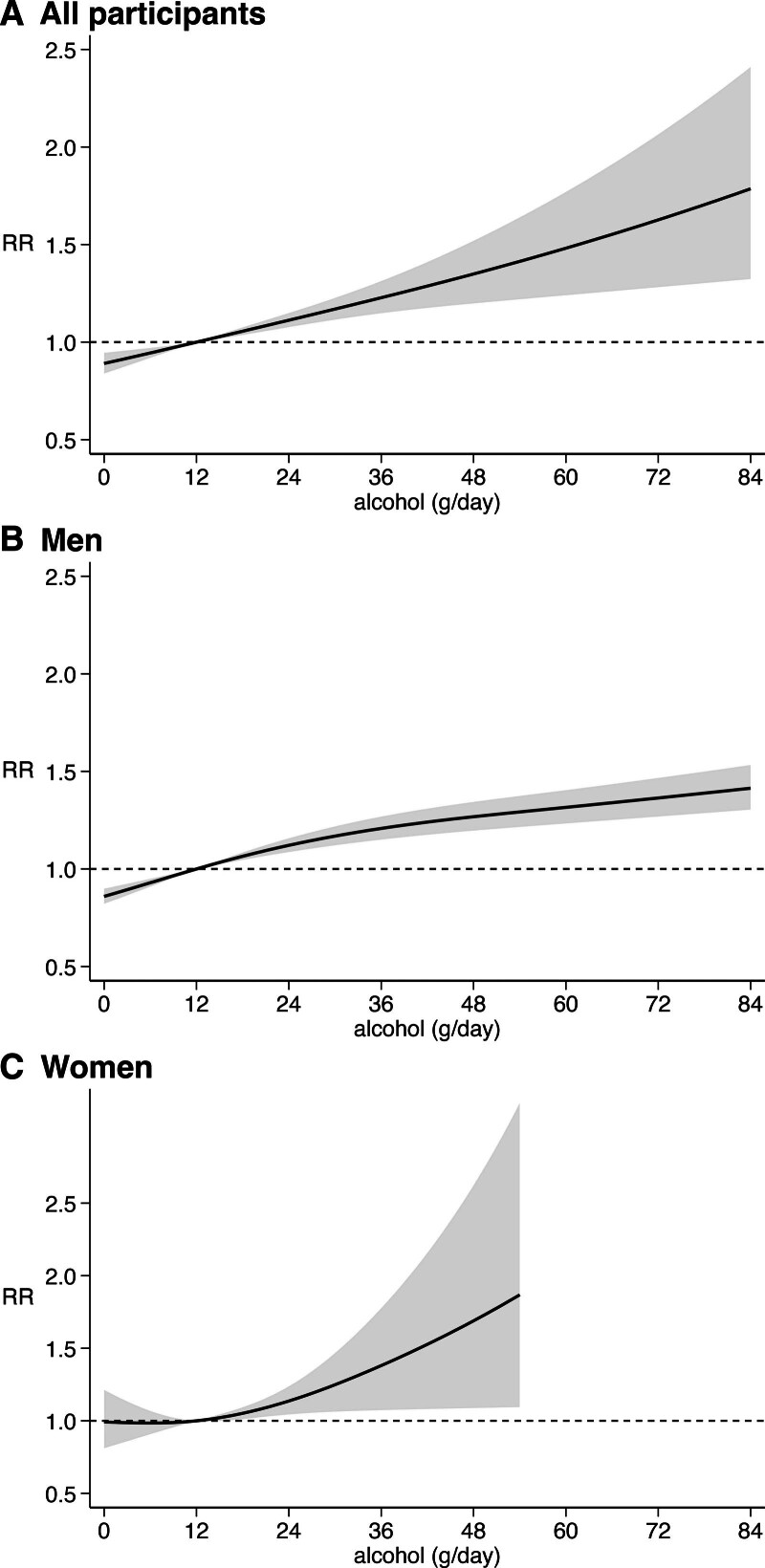
**Dose-response meta-analysis of the risk ratio (RR) of hypertension according to alcohol consumption (g/day).** Analysis presented in (**A**) all study participants (22 studies^9,13,22,24–42^), in (**B**) men (18 studies^9,22–24,26–33,35–39,41^), and in (**C**) women (12 studies^9,23,26,27,29,31,36,38–42^). Overall spline curve (black solid line) with 95% confidence limits (gray area).

In Figure 2, we present the results of the dose-response meta-analysis in the overall population and men and women. In the overall analysis (22 studies), there was a linear positive association between alcohol consumption and incidence of hypertension above an alcohol intake of 12 g/d, with RRs using 12 g/d as reference of 0.89 (95% CI, 0.84–0.94), 1.11 (95% CI, 1.07–1.15), 1.22 (95% CI, 1.14–1.30), 1.33 (95% CI, 1.18–1.49) at 0, 24, 36, and 48 g/d of alcohol consumption, respectively. In sex-stratified analyses, men (18 studies) demonstrated a linear positive association between alcohol intake and risk of hypertension, steeper in the range 0 to 36 g/d, but the risk flattened at higher level of intake, with RRs using 12 g/d as reference of 0.86 (95% CI, 0.82–0.90), 1.12 (95% CI, 1.09–1.16), 1.21 (95% CI, 1.15–1.27), and 1.27 (95% CI, 1.20–1.35) at 0, 24, 36, and 48 g/d of alcohol consumption, respectively (Figure 2). In women (12 studies), for whom risk estimates were statistically unstable compared with men, there was no indication of an increased risk of hypertension up to 12 g/d of alcohol consumption, while the risk started to increase at higher intakes of alcohol with a steeper slope at increasing levels of consumption and RRs, using 12 g/d as the reference, of 0.99 (95% CI, 0.81–1.21), 1.14 (95% CI, 1.04–1.24), 1.38 (95% CI, 1.07–1.78), and 1.69 (95% CI, 1.09–2.62) at 0, 24, 36 and 48 g/d of alcohol consumption, respectively. Sensitivity analysis excluding studies at high risk of bias did not substantially change the findings (Figure S4). Exclusion of studies using cutoffs for definition of hypertension higher than BP ≥140/90 mm Hg or not reporting them yielded similar results (Figure S5). Conversely, exclusion of studies that did not adjust for smoking yielded similar results although the association was generally less steep with lower RRs compared with the analysis considering all studies (Figure S6). The stratified analyses by duration of follow-up (<20 and ≥20 years) yielded substantially similar patterns for the association, although it was less steep for studies with a longer duration of follow-up (Figure S7).

**Figure 3.**
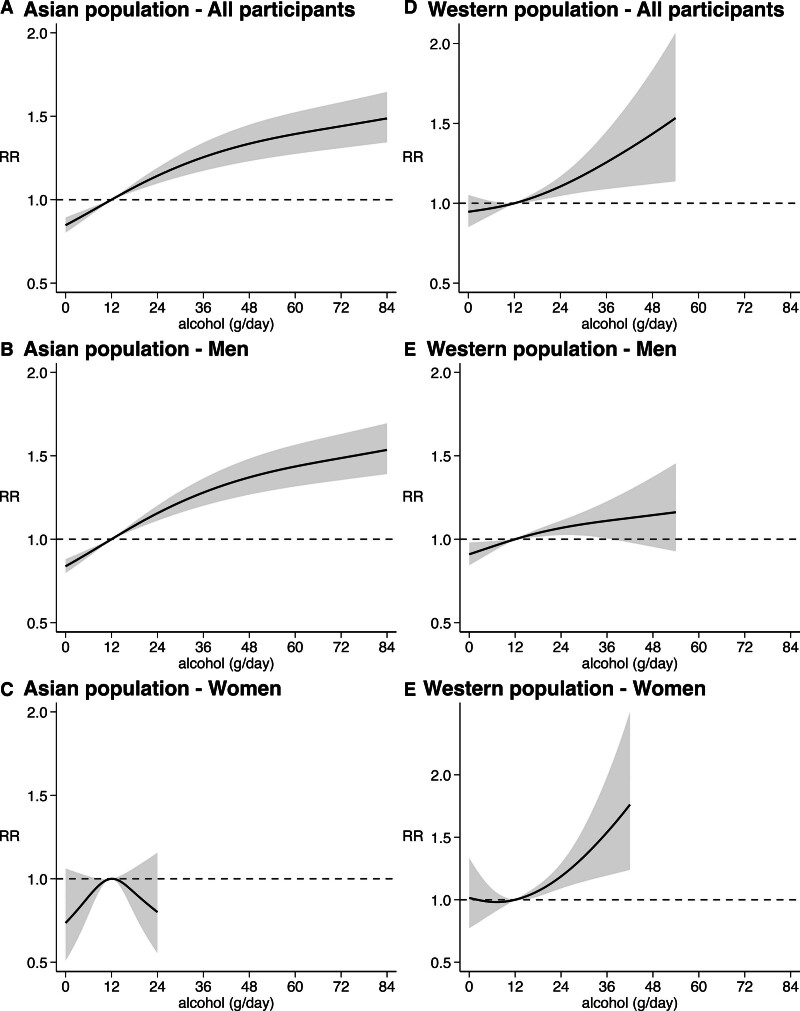
**Dose-response meta-analysis of the risk ratio (RR) of hypertension according to alcohol consumption (g/day) divided by region and sex.** Analysis presented by Asian and Western region and sex (Asian [**A**] n=11 studies overall,^9,13,28–30,32,33,35–37,41^ [**B**] n=11 studies in men,^9,23,28–30,32,33,35–37,41^ and [**C**] n=3 in women,^9,29,36^ and Western, (**D**) n=11 studies overall,^22,24–27,31,34,38–40,42^ (**E**) n=7 studies in men,^22,24,26,27,31,38,39^ and (**F**) n=7 in women^26,27,31,38–40,42^). Overall spline curve (black solid line) with 95% confidence limits (gray area).

In the subgroup analysis of the 11 studies conducted in Asian populations (Figure 3), we identified an almost linear positive association between alcohol intake and the risk of hypertension in the range of 0 to 36 g/d of alcohol intake, with a less steep increase in risk at the highest level of exposure. Using 12 g/d as the reference group, RRs were 0.85 (95% CI, 0.80–0.89), 1.14 (95% CI, 1.10–1.19), 1.25 (95% CI, 1.17–1.34), and 1.34 (95% CI, 1.23–1.45) at 0, 24, 36, and 48 g/d of alcohol consumption, respectively. In contrast, pooling of the 11 studies conducted in Western populations identified usual alcohol intake as having little association with risk of hypertension for intakes up to 12 gr per day, while at higher intakes a positive association emerged, with an upward inflection in the shape of the curve. In these Western populations, using 12 g/d as the reference group RRs were 0.95 (95% CI, 0.85–1.05), 1.11 (95% CI, 1.05–1.17), 1.26 (95% CI, 1.09–1.45), and 1.43 (95% CI, 1.12–1.84) at 0, 24, 36, and 48 g/d of alcohol consumption, respectively. Sex-stratified analyses in the Asian and Western populations (Figure 3) showed rather different shapes. In men, the association between alcohol intake and risk of hypertension was roughly similar between Asian and Western populations (11 and 7 studies, respectively) though with a steeper increase and higher RRs in the formers. In women, no association emerged in Asian population (though based on 3 studies only, and limited to a narrow range of intake), while in Western women (7 studies) a positive association started to emerge approaching 24 g/d of alcohol (corresponding to 2 drinks) and showed a steep increase above that amount.

One study only^31^ reported data for Hispanics and half of the studies carried out in Western population did not specify ethnicity. Stratified analysis by ethnicity was therefore only feasible in a small number of studies for Black (4 studies) and White (4 studies) populations.

**Figure 4.**
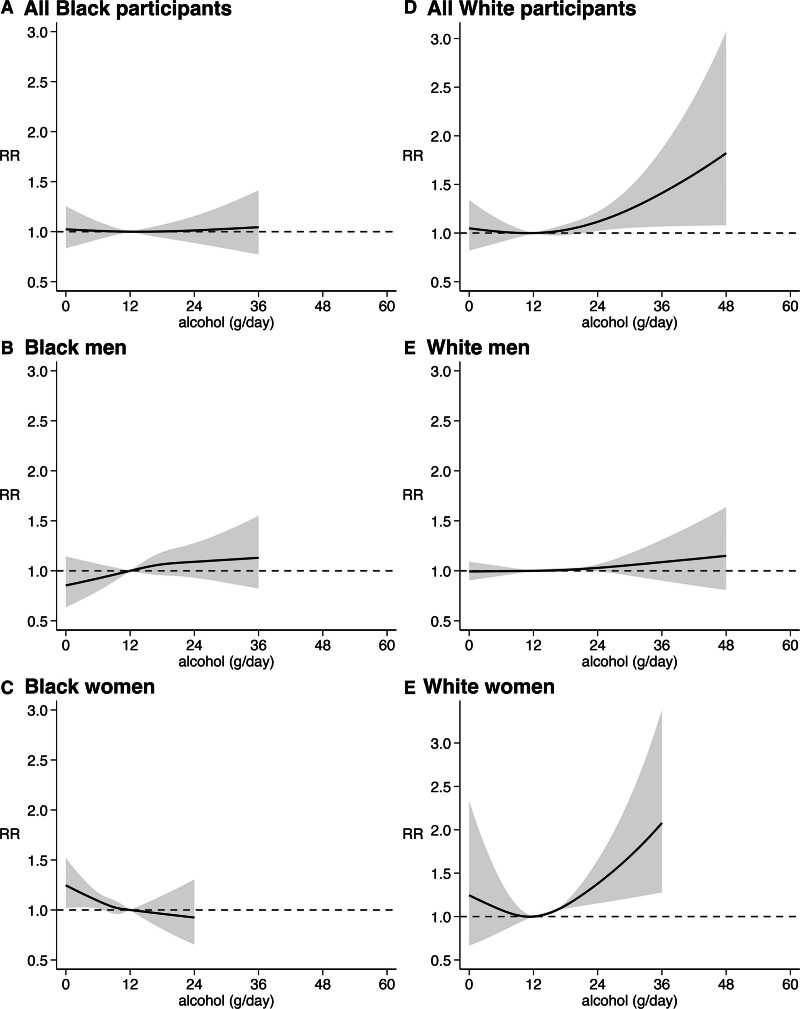
**Dose-response meta-analysis of the risk ratio (RR) of hypertension according to alcohol consumption (g/day) divided by ethnicity and sex.** Analysis presented stratified by Black and White populations and by sex (Black population, [**A**] n=4 studies overall,^25–27,31^ [**B**] n=3 in men,^26,27,31^ and [**C**] n=3 in women^26,27,31^; White population, [**D**] n=5 studies overall,^24,26,27,31,42^ [**E**] n=4 studies in men,^24,26,27,31^ and [**F**] n=4 studies in women^26,27,31,42^). Overall spline curve (black solid line) with 95% confidence limits (gray area).

The association between alcohol consumption in Black population showed no overall increase in the risk of hypertension, while in sex-specific analysis a weak positive association was apparent in men (3 studies), while in women a higher risk emerged only in those consuming little or no alcohol (3 studies; Figure 4).

In White population, the was no association between alcohol intake and risk of hypertension at low levels of consumption (<12 g/d), while risk considerably and steeply increased above 24 g/d. In sex-specific analysis, such association was only partially confirmed in men (4 studies), with an apparent increased risk above 24 g/d. Conversely, in women, a considerably and steeply increasing risk emerged above intakes of 12 g/d (4 studies), and also abstainers showed an increased risk, similar to that noted in Black women.

Sensitivity analysis investigating study-specific curves showed high variation across studies, especially in the overall and men-restricted analyses (Figure S8). Assessment of publication bias through funnel plots analysis showed little evidence of small-study bias, as suggested by a substantially symmetrical distribution in overall and sex-specific analyses (Figure S9).

## DISCUSSION

In this systematic review and meta-analysis, we found that increasing alcohol consumption was positively and almost entirely linearly associated with the risk of new-onset hypertension in the overall analysis, but in women the excess risk was apparent only above an alcohol intake of about 12 g/d. In addition, the shape of the increasing RR in men and women was different, with a trend toward an attenuation of the increase in risk in men at higher levels of alcohol consumption, while in women the opposite was true, with a dose-response curve that showed a trend towards a steeper pattern at higher levels of alcohol consumption. This suggests that sex acts as an effect-modifier for the association between alcohol intake and risk of hypertension, and that low levels of alcohol consumption may not increase the risk of hypertension in women, while at higher levels of alcohol intake the risk of hypertension appears to be higher than in men. Therefore, based on our analysis, a moderate to high usual intake of alcohol seems to be a risk factor for hypertension in both men and women, with a stepper slope in women. In contrast, at low levels of alcohol intake, an increased risk of hypertension may only apply in men.

The association between alcohol consumption and hypertension is especially important because of the high prevalence of alcohol consumption above a light intake in many countries and the well-demonstrated association between hypertension and adverse effects, including risk of cardiovascular disease, cognitive decline, and kidney damage.^44–47^ Our findings have implications for public health and recommendations related to the safety of alcohol consumption, both for men (in whom, any consumption may be detrimental, but particularly a high consumption) and women (in whom 1–1.5 drinks a day may not to be related to an excess risk of hypertension). In a previous meta-analysis, a similar alcohol intake threshold for risk of hypertension was identified in women while no inflection point and therefore no safe range of intake was identified in men.^8^

Our findings are in contrast with previous reports suggesting that light to moderate alcohol consumption does not adversely affect the risk of hypertension, and may even decrease it depending on sex and ethnicity,^26,48^ since the RR pattern we computed did not suggest any beneficial effect of low levels of alcohol consumption compared with no consumption and especially with a high consumption for risk of hypertension. In a previous meta-analysis, based on a smaller number of studies, there was a slight indication of an inverse association with the risk of hypertension in women consuming small amounts of alcohol, that is, 1 to 2 drinks/day (RR, 0.95 [95% CI, 0.89–1.02]).^8^

In our meta-analysis, the increased risk of hypertension with an intake of 12 g/d in men, and 24 g/d in women was modest in clinical terms (+10% to 12%) and less than that noted for other exposures such as excessive sodium consumption.^49^ However, the modest hypertension risk associations noted at low levels of alcohol consumption may be relevant for population health, supporting public health recommendations to avoid or limit alcohol consumption as much as possible to prevent the increase of BP and onset of hypertension.

With reference to subgroup analyses by continent and ethnicity, we found an indication for a threshold of risk of hypertension in Westerners, but not in Asians, and evidence of effect-modification by sex in both populations. In fact, there was an indication for a lower susceptibility to an adverse effect of alcohol consumption on hypertension risk in Western compared with Asian men, but this was not true for women. In the latter, clear evidence of increased risk emerged only in Western women and above the approximate threshold of 12 g/d, although the limited number of studies hampers the interpretation of these results. The differences across ethnic groups could be explained by genetic factors and particularly by a lower expression of ADH (alcohol dehydrogenase) and aldehyde dehydrogenase (ALDH) in Asians compared with Westerners.^50,51^

The findings of the study suggest a possibly lower susceptibility to the effect of alcohol consumption in Black population, despite the unfortunately limited number of studies that reported specific analyses in Black and White populations, particularly for sex-specific analyses, and the related uncertainties. Both in Black and White populations there was little evidence for a detrimental effects of increased alcohol consumption up to 12 to 24 g/d in the overall population and both sexes, though in White population a higher consumption started to be associated with an increased hypertension risk, mainly due to a steep increase in women. A tendency for a weaker association between alcohol consumption and BP in Black population has long been noted,^52^ and a lower susceptibility has been found in Black population for other health conditions and it has been ascribed to genetic influences, especially to APOL1 (apoliprotein L1) genotype.^53^ Unfortunately, no genetic data were available in the dataset we studied. In addition, residual confounding might still explain the differences we identified for the alcohol-hypertension risks in Black and White populations.^54^ Finally, it is worth noting that in the analysis stratified for both sex and ethnicity, Black and White women not consuming any alcohol exhibited a slightly increased risk of hypertension as compared with low consumers, though this finding was based on a small number of studies, thus it needs to be confirmed by additional investigations.

Risk of bias in the included studies appeared to have little impact on the dose-response estimates, as demonstrated by our analysis restricted to studies with low to moderate risk of bias. In addition, the overall analysis did not appear to be driven by the results of a specific study, and such consistency supports the validity of the overall meta-analysis and the shape of association we obtained when pooling the individual studies.

There is biological plausibility for the detrimental effect of alcohol on the risk of hypertension. One potential mechanism for an alcohol effect on BP and risk of hypertension is stimulation of the renin-angiotensin-aldosterone system^55^ with a resultant increase in angiotensin II and plasma cortisol levels.^56^ Additional possibilities are sympathetic nervous system stimulation due to increases in noradrenaline levels,^57–61^ decrease of baroreceptor sensitivity,^62–64^ and an increase of intracellular calcium leading to blood vessel constriction,^1,65^ Hormonal factors might also explain the different associations between alcohol intake and the risk of hypertension in men and women particularly at low levels of intake, given the relevance of ovarian hormones and of testosterone in BP regulation.^66–69^

The use of baseline assessment of alcohol consumption is a limitation of our review since we could not rule out the risk of exposure misclassification and change over time of drinking habits due to the limited number of studies that provided alcohol consumption reports during follow-up and the inability to consider study duration within our model. However, 1 study^25^ that investigated the risk of hypertension in participants who changed their alcohol consumption, reported that those who discontinued alcohol consumption experienced no substantial change in their BP levels and no increase in hypertension risk. Conversely, those who continued or initiated alcohol consumption during follow-up showed an increased risk of hypertension.

In our analysis, we used aggregated rather than individual participant data for our analyses. Obtaining individual data would likely have been difficult if not impossible for the studies selected. In addition, there is evidence that using the 1-stage approach for the calculation of risk ratio estimates generally allows a good approximation of the estimates that are obtained by the pooling of individual data.^15^ Another limitation inherent in our study database was the relatively small number of studies that reported findings by ethnicity, precluding the implementation of stratified dose-response analysis in Hispanics and highly limiting the precision of our risk estimates in Black and White populations, particularly in sex-stratified analyses. Finally, our findings may not apply to ranges of alcohol consumption outside those reported in the included studies, generally in the range of 0 to 84 g/d for men and even lower for women, such as in individuals characterized by extremely high amounts of average alcohol consumption, or binge consumption patterns.

## ARTICLE INFORMATION

### Sources of Funding

This project was supported by grant Fondo di Ateneo per la Ricerca (FAR) 2023 from the University of Modena and Reggio Emilia. T. Filippini was supported by grants Progetti di Rilevante Interesse Nazionale (PRIN) 2022 (no. 2022MHMRPR) and Progetti di Rilevante Interesse Nazionale (PRIN) 2022 PNRR (no. P20229KSXB) from the Italian Ministry of University and Research funded by European Union – Next Generation EU. P.K. Whelton was supported by National Institutes of Health Centers of Biomedical Excellence grant (P20GM109036).

### Disclosures

None.

## Supplementary Material

## References

[1] Tasnim S Tang C Musini VM Wright JM. Effect of alcohol on blood pressure. Cochrane Database Syst Rev. 2020;7:CD012787. doi: 10.1002/14651858.CD012787.pub2 32609894 10.1002/14651858.CD012787.pub2 PMC8130994

[2] World Health Organization. Alcohol. 2023. Accessed February 24, 2024. https://www.who.int/health-topics/alcohol#tab=tab_1

[3] Kalinowski A Humphreys K. Governmental standard drink definitions and low-risk alcohol consumption guidelines in 37 countries. Addiction. 2016;111:1293–1298. doi: 10.1111/add.13341 27073140 10.1111/add.13341

[4] Mongan D Long J. Standard drink measures in Europe Peoples’ understanding of standard drinks and their use in drinking guidelines, alcohol surveys and labelling. 2015. Accessed February 24, 2024. https://www.rarha.eu/Resources/Deliverables/Lists/Deliverables/Attachments/14/WP5%20Background%20paper%20Standard%20drink%20measures%20HRB.pdf

[5] Lucas DL Brown RA Wassef M Giles TD. Alcohol and the cardiovascular system: research challenges and opportunities. J Am Coll Cardiol. 2005;45:1916–1924. doi: 10.1016/j.jacc.2005.02.075 15963387 10.1016/j.jacc.2005.02.075

[6] Di Federico S Filippini T Whelton PK Cecchini M Iamandii I Boriani G Vinceti M. Alcohol intake and blood pressure levels: a dose-response meta-analysis of nonexperimental cohort studies. Hypertension. 2023;80:1961–1969. doi: 10.1161/HYPERTENSIONAHA.123.21224 37522179 10.1161/HYPERTENSIONAHA.123.21224 PMC10510850

[7] Fuchs FD Fuchs SC. The effect of alcohol on blood pressure and hypertension. Curr Hypertens Rep. 2021;23:42. doi: 10.1007/s11906-021-01160-7 34762198 10.1007/s11906-021-01160-7

[8] Roerecke M Tobe SW Kaczorowski J Bacon SL Vafaei A Hasan OSM Krishnan RJ Raifu AO Rehm J. Sex-specific associations between alcohol consumption and incidence of hypertension: a systematic review and meta-analysis of cohort studies. J Am Heart Assoc. 2018;7:e008202. doi: 10.1161/JAHA.117.008202 29950485 10.1161/JAHA.117.008202 PMC6064910

[9] Yoo MG Park KJ Kim HJ Jang HB Lee HJ Park SI. Association between alcohol intake and incident hypertension in the Korean population. Alcohol. 2019;77:19–25. doi: 10.1016/j.alcohol.2018.09.002 30236891 10.1016/j.alcohol.2018.09.002

[10] Page MJ McKenzie JE Bossuyt PM Boutron I Hoffmann TC Mulrow CD Shamseer L Tetzlaff JM Akl EA Brennan SE. The PRISMA 2020 statement: an updated guideline for reporting systematic reviews. BMJ. 2021;372:n71. doi: 10.1136/bmj.n71 33782057 10.1136/bmj.n71 PMC8005924

[11] Morgan RL Whaley P Thayer KA Schunemann HJ. Identifying the PECO: a framework for formulating good questions to explore the association of environmental and other exposures with health outcomes. Environ Int. 2018;121:1027–1031. doi: 10.1016/j.envint.2018.07.015 30166065 10.1016/j.envint.2018.07.015 PMC6908441

[12] US CDC. Alcohol and public health. 2023. Accessed February 24, 2024. https://www.cdc.gov/alcohol/

[13] Qiu W Cai A Li L Feng Y. Longitudinal trajectories of alcohol consumption with all-cause mortality, hypertension, and blood pressure change: results from CHNS Cohort, 1993-2015. Nutrients. 2022;14:5073. doi: 10.3390/nu14235073 36501103 10.3390/nu14235073 PMC9739068

[14] Morgan RL Thayer KA Santesso N Holloway AC Blain R Eftim SE Goldstone AE Ross P Ansari M Akl EA; GRADE Working Group. A risk of bias instrument for non-randomized studies of exposures: a users’ guide to its application in the context of GRADE. Environ Int. 2019;122:168–184. doi: 10.1016/j.envint.2018.11.004 30473382 10.1016/j.envint.2018.11.004 PMC8221004

[15] Crippa A Discacciati A Bottai M Spiegelman D Orsini N. One-stage dose-response meta-analysis for aggregated data. Stat Methods Med Res. 2019;28:1579–1596. doi: 10.1177/0962280218773122 29742975 10.1177/0962280218773122

[16] Harrell FE. Regression modeling strategies. New York, NY, USA: Springer; 2001

[17] Veneri F Vinceti M Generali L Giannone ME Mazzoleni E Birnbaum LS Consolo U Filippini T. Fluoride exposure and cognitive neurodevelopment: systematic review and dose-response meta-analysis. Environ Res. 2023;221:115239. doi: 10.1016/j.envres.2023.115239 36639015 10.1016/j.envres.2023.115239

[18] Filippini T Wise LA Vinceti M. Cadmium exposure and risk of diabetes and prediabetes: a systematic review and dose-response meta-analysis. Environ Int. 2022;158:106920. doi: 10.1016/j.envint.2021.106920 34628255 10.1016/j.envint.2021.106920

[19] Iamandii I De Pasquale L Giannone ME Veneri F Generali L Consolo U Birnbaum LS Castenmiller J Halldorsson TI Filippini T. Does fluoride exposure affect thyroid function? A systematic review and dose-response meta-analysis. Environ Res. 2024;242:117759. doi: 10.1016/j.envres.2023.117759 38029816 10.1016/j.envres.2023.117759

[20] Murad MH Verbeek J Schwingshackl L Filippini T Vinceti M Akl EA Morgan RL Mustafa RA Zeraatkar D Senerth E; GRADE Working Group. GRADE guidance 38: updated guidance for rating up certainty of evidence due to a dose-response gradient. J Clin Epidemiol. 2023;164:45–53. doi: 10.1016/j.jclinepi.2023.09.011 37777140 10.1016/j.jclinepi.2023.09.011

[21] Villoz F Filippini T Ortega N Kopp-Heim D Voortman T Blum MR Del Giovane C Vinceti M Rodondi N Chocano-Bedoya PO. Dairy intake and risk of cognitive decline and dementia: a systematic review and dose-response meta-analysis of prospective studies. Adv Nutr. 2024;15:100160. doi: 10.1016/j.advnut.2023.100160 38043604 10.1016/j.advnut.2023.100160 PMC10788406

[22] Niskanen L Laaksonen DE Nyyssonen K Punnonen K Valkonen VP Fuentes R Tuomainen TP Salonen R Salonen JT. Inflammation, abdominal obesity, and smoking as predictors of hypertension. Hypertension. 2004;44:859–865. doi: 10.1161/01.HYP.0000146691.51307.84 15492131 10.1161/01.HYP.0000146691.51307.84

[23] Bai G Zhang J Zhao C Wang Y Qi Y Zhang B. Adherence to a healthy lifestyle and a DASH-style diet and risk of hypertension in Chinese individuals. Hypertens Res. 2017;40:196–202. doi: 10.1038/hr.2016.119 27604345 10.1038/hr.2016.119

[24] Banda JA Clouston K Sui X Hooker SP Lee CD Blair SN. Protective health factors and incident hypertension in men. Am J Hypertens. 2010;23:599–605. doi: 10.1038/ajh.2010.26 20224555 10.1038/ajh.2010.26

[25] Curtis AB James SA Strogatz DS Raghunathan TE Harlow S. Alcohol consumption and changes in blood pressure among African Americans. The Pitt county study. Am J Epidemiol. 1997;146:727–733. doi: 10.1093/oxfordjournals.aje.a009348 9366620 10.1093/oxfordjournals.aje.a009348

[26] Fuchs FD Chambless LE Whelton PK Nieto FJ Heiss G. Alcohol consumption and the incidence of hypertension: the atherosclerosis risk in communities study. Hypertension. 2001;37:1242–1250. doi: 10.1161/01.hyp.37.5.1242 11358935 10.1161/01.hyp.37.5.1242

[27] Halanych JH Safford MM Kertesz SG Pletcher MJ Kim YI Person SD Lewis CE Kiefe CI. Alcohol consumption in young adults and incident hypertension: 20-year follow-up from the coronary artery risk development in young adults study. Am J Epidemiol. 2010;171:532–539. doi: 10.1093/aje/kwp417 20118194 10.1093/aje/kwp417 PMC2842215

[28] Im PK Wright N Yang L Chan KH Chen Y Guo Y Du H Yang X Avery D Wang S; China Kadoorie Biobank Collaborative Group. Alcohol consumption and risks of more than 200 diseases in Chinese men. Nat Med. 2023;29:1476–1486. doi: 10.1038/s41591-023-02383-8 37291211 10.1038/s41591-023-02383-8 PMC10287564

[29] Jung S Kim MK Shin J Lee N Woo HW Choi BY Shin MH Shin DH Lee YH. Positive association of alcohol consumption with incidence of hypertension in adults aged 40 years and over: use of repeated alcohol consumption measurements. Clin Nutr. 2020;39:3125–3131. doi: 10.1016/j.clnu.2020.01.020 32044137 10.1016/j.clnu.2020.01.020

[30] Lee SH Kim YS Sunwoo S Huh BY. A retrospective cohort study on obesity and hypertension risk among Korean adults. J Korean Med Sci. 2005;20:188–195. doi: 10.3346/jkms.2005.20.2.188 15831985 10.3346/jkms.2005.20.2.188 PMC2808590

[31] Lui CK Kerr WC Li L Mulia N Ye Y Williams E Greenfield TK Lown EA. Lifecourse drinking patterns, hypertension, and heart problems among U.S. adults. Am J Prev Med. 2020;58:386–395. doi: 10.1016/j.amepre.2019.10.018 31928761 10.1016/j.amepre.2019.10.018 PMC7176748

[32] Nagao T Nogawa K Sakata K Morimoto H Morita K Watanabe Y Suwazono Y. Effects of alcohol consumption and smoking on the onset of hypertension in a long-term longitudinal study in a male workers’ cohort. Int J Environ Res Public Health. 2021;18:11781. doi: 10.3390/ijerph182211781 34831535 10.3390/ijerph182211781 PMC8619602

[33] Nakanishi N Yoshida H Nakamura K Suzuki K Tatara K. Alcohol consumption and risk for hypertension in middle-aged Japanese men. J Hypertens. 2001;19:851–855. doi: 10.1097/00004872-200105000-00003 11393666 10.1097/00004872-200105000-00003

[34] Nunez-Cordoba JM Martinez-Gonzalez MA Bes-Rastrollo M Toledo E Beunza JJ Alonso A. Alcohol consumption and the incidence of hypertension in a Mediterranean cohort: the SUN study. Rev Esp Cardiol. 2009;62:633–641. doi: 10.1016/s1885-5857(09)72227-3 19480759 10.1016/s1885-5857(09)72227-3

[35] Ohmori S Kiyohara Y Kato I Kubo M Tanizaki Y Iwamoto H Nakayama K Abe I Fujishima M. Alcohol intake and future incidence of hypertension in a general Japanese population: the Hisayama study. Alcohol Clin Exp Res. 2002;26:1010–1016. doi: 10.1097/01.ALC.0000021147.31338.C2 12170111 10.1097/01.ALC.0000021147.31338.C2

[36] Okubo Y Sairenchi T Irie F Yamagishi K Iso H Watanabe H Muto T Tanaka K Ota H. Association of alcohol consumption with incident hypertension among middle-aged and older Japanese population: the Ibarakai Prefectural Health Study (IPHS). Hypertension. 2014;63:41–47. doi: 10.1161/HYPERTENSIONAHA.113.01585 24126168 10.1161/HYPERTENSIONAHA.113.01585

[37] Peng M Wu S Jiang X Jin C Zhang W; Kailuan Cardiovascular Survey Group. ., Kailuan Cardiovascular Survey G. Long-term alcohol consumption is an independent risk factor of hypertension development in northern China: evidence from Kailuan study. J Hypertens. 2013;31:2342–2347. doi: 10.1097/HJH.0b013e3283653999 24029874 10.1097/HJH.0b013e3283653999

[38] Saremi A Hanson RL Tulloch-Reid M Williams DE Knowler WC. Alcohol consumption predicts hypertension but not diabetes. J Stud Alcohol. 2004;65:184–190. doi: 10.15288/jsa.2004.65.184 15151348 10.15288/jsa.2004.65.184

[39] Sesso HD Cook NR Buring JE Manson JE Gaziano JM. Alcohol consumption and the risk of hypertension in women and men. Hypertension. 2008;51:1080–1087. doi: 10.1161/HYPERTENSIONAHA.107.104968 18259032 10.1161/HYPERTENSIONAHA.107.104968

[40] Thadhani R Camargo CA Jr Stampfer MJ Curhan GC Willett WC Rimm EB. Prospective study of moderate alcohol consumption and risk of hypertension in young women. Arch Intern Med. 2002;162:569–574. doi: 10.1001/archinte.162.5.569 11871925 10.1001/archinte.162.5.569

[41] Wang Y Yao Y Chen Y Zhou J Wu Y Fu C Wang N Liu T Xu K. Association between drinking patterns and incident hypertension in Southwest China. Int J Environ Res Public Health. 2022;19:3801. doi: 10.3390/ijerph19073801 35409487 10.3390/ijerph19073801 PMC8997936

[42] Witteman JC Willett WC Stampfer MJ Colditz GA Kok FJ Sacks FM Speizer FE Rosner B Hennekens CH. Relation of moderate alcohol consumption and risk of systemic hypertension in women. Am J Cardiol. 1990;65:633–637. doi: 10.1016/0002-9149(90)91043-6 2309634 10.1016/0002-9149(90)91043-6

[43] Jung S Kim MK Shin J Choi BY Lee YH Shin DH Shin MH. The longitudinal associations between trajectory of and quantity of alcohol consumption and subsequent changes in blood pressure levels among non-hypertensive adults. Br J Nutr. 2021;126:1380–1388. doi: 10.1017/S0007114521000088 33441197 10.1017/S0007114521000088

[44] Burnier M Damianaki A. Hypertension as cardiovascular risk factor in chronic kidney disease. Circ Res. 2023;132:1050–1063. doi: 10.1161/CIRCRESAHA.122.321762 37053276 10.1161/CIRCRESAHA.122.321762

[45] Carey RM Wright JT Jr Taler SJ Whelton PK. Guideline-driven management of hypertension: an evidence-based update. Circ Res. 2021;128:827–846. doi: 10.1161/CIRCRESAHA.121.318083 33793326 10.1161/CIRCRESAHA.121.318083 PMC8034801

[46] Kohagura K. The public health impact of hypertension and diabetes: a powerful tag team for the development of chronic kidney disease. Hypertens Res. 2023;46:339–340. doi: 10.1038/s41440-022-01114-9 36474030 10.1038/s41440-022-01114-9

[47] Mulligan MD Murphy R Reddin C Judge C Ferguson J Alvarez-Iglesias A McGrath ER O’Donnell MJ. Population attributable fraction of hypertension for dementia: global, regional, and national estimates for 186 countries. EClinicalMedicine. 2023;60:102012. doi: 10.1016/j.eclinm.2023.102012 37261323 10.1016/j.eclinm.2023.102012 PMC10227413

[48] Huntgeburth M Ten Freyhaus H Rosenkranz S. Alcohol consumption and hypertension. Curr Hypertens Rep. 2005;7:180–185. doi: 10.1007/s11906-005-0007-2 15913491 10.1007/s11906-005-0007-2

[49] Filippini T Malavolti M Whelton PK Naska A Orsini N Vinceti M. Blood pressure effects of sodium reduction: dose-response meta-analysis of experimental studies. Circulation. 2021;143:1542–1567. doi: 10.1161/CIRCULATIONAHA.120.050371 33586450 10.1161/CIRCULATIONAHA.120.050371 PMC8055199

[50] Chrostek L Jelski W Szmitkowski M Puchalski Z. Gender-related differences in hepatic activity of alcohol dehydrogenase isoenzymes and aldehyde dehydrogenase in humans. J Clin Lab Anal. 2003;17:93–96. doi: 10.1002/jcla.10076 12696080 10.1002/jcla.10076 PMC6807748

[51] Kim HY Choi CK Kweon SS Lee YH Nam HS Park KS Ryu SY Choi SW Shin MH. Effect modification of acetaldehyde dehydrogenase 2 rs671 polymorphism on the association between alcohol intake and blood pressure: the Dong-gu study. J Korean Med Sci. 2020;35:e14. doi: 10.3346/jkms.2020.35.e14 32141245 10.3346/jkms.2020.35.e14 PMC7061145

[52] Klatsky AL Friedman GD Armstrong MA. The relationships between alcoholic beverage use and other traits to blood pressure: a new Kaiser permanente study. Circulation. 1986;73:628–636. doi: 10.1161/01.cir.73.4.628 3948365 10.1161/01.cir.73.4.628

[53] Chen TK Katz R Estrella MM Post WS Kramer H Rotter JI Tayo B Mychaleckyj JC Wassel CL Peralta CA. Association of APOL1 genotypes with measures of microvascular and endothelial function, and blood pressure in MESA. J Am Heart Assoc. 2020;9:e017039. doi: 10.1161/JAHA.120.017039 32851884 10.1161/JAHA.120.017039 PMC7660790

[54] Liu K Ruth KJ Flack JM Jones-Webb R Burke G Savage PJ Hulley SB. Blood pressure in young blacks and whites: relevance of obesity and lifestyle factors in determining differences. The CARDIA study. Coronary artery risk development in young adults. Circulation. 1996;93:60–66. doi: 10.1161/01.cir.93.1.60 8616942 10.1161/01.cir.93.1.60

[55] Puddey IB Vandongen R Beilin LJ Rouse IL. Alcohol stimulation of renin release in man: its relation to the hemodynamic, electrolyte, and sympatho-adrenal responses to drinking. J Clin Endocrinol Metab. 1985;61:37–42. doi: 10.1210/jcem-61-1-37 3889040 10.1210/jcem-61-1-37

[56] Jenkins JS Connolly J. Adrenocortical response to ethanol in man. Br Med J. 1968;2:804–805. doi: 10.1136/bmj.2.5608.804 5656299 10.1136/bmj.2.5608.804 PMC1991619

[57] Barden AE Croft KD Beilin LJ Phillips M Ledowski T Puddey IB. Acute effects of red wine on cytochrome P450 eicosanoids and blood pressure in men. J Hypertens. 2013;31:2195–202; discussion 2202. doi: 10.1097/HJH.0b013e328364a27f 24096258 10.1097/HJH.0b013e328364a27f

[58] Grassi GM Somers VK Renk WS Abboud FM Mark AL. Effects of alcohol intake on blood pressure and sympathetic nerve activity in normotensive humans: a preliminary report. J Hypertens Suppl. 1989;7:S20–S21. doi: 10.1097/00004872-198900076-00007 2632716 10.1097/00004872-198900076-00007

[59] Randin D Vollenweider P Tappy L Jequier E Nicod P Scherrer U. Suppression of alcohol-induced hypertension by dexamethasone. N Engl J Med. 1995;332:1733–1737. doi: 10.1056/NEJM199506293322601 7760888 10.1056/NEJM199506293322601

[60] Russ R Abdel-Rahman AR Wooles WR. Role of the sympathetic nervous system in ethanol-induced hypertension in rats. Alcohol. 1991;8:301–307. doi: 10.1016/0741-8329(91)90433-w 1872991 10.1016/0741-8329(91)90433-w

[61] Zhang X Abdel-Rahman AA Wooles WR. Impairment of baroreceptor reflex control of heart rate but not sympathetic efferent discharge by central neuroadministration of ethanol. Hypertension. 1989;14:282–292. doi: 10.1161/01.hyp.14.3.282 2767759 10.1161/01.hyp.14.3.282

[62] Abdel-Rahman AR Dar MS Wooles WR. Effect of chronic ethanol administration on arterial baroreceptor function and pressor and depressor responsiveness in rats. J Pharmacol Exp Ther. 1985;232:194–201 4038417

[63] Carretta R Fabris B Bardelli M Muiesan S Fischetti F Cesanelli R Pizzolitto A Bianchetti A Campanacci L. Acute effects of intravenous infusions of alcohol on baroreceptor sensitivity in essential hypertension. Cardiovasc Res. 1988;22:226–230. doi: 10.1093/cvr/22.3.226 3167946 10.1093/cvr/22.3.226

[64] Rupp H Brilla CG Maisch B. [Hypertension and alcohol: central and peripheral mechanisms]. Herz. 1996;21:258–264 8805006

[65] Husain K Ansari RA Ferder L. Alcohol-induced hypertension: mechanism and prevention. World J Cardiol. 2014;6:245–252. doi: 10.4330/wjc.v6.i5.245 24891935 10.4330/wjc.v6.i5.245 PMC4038773

[66] Colafella KMM Denton KM. Sex-specific differences in hypertension and associated cardiovascular disease. Nat Rev Nephrol. 2018;14:185–201. doi: 10.1038/nrneph.2017.189 29380817 10.1038/nrneph.2017.189

[67] Gerdts E Sudano I Brouwers S Borghi C Bruno RM Ceconi C Cornelissen V Dievart F Ferrini M Kahan T. Sex differences in arterial hypertension. Eur Heart J. 2022;43:4777–4788. doi: 10.1093/eurheartj/ehac470 36136303 10.1093/eurheartj/ehac470 PMC9726450

[68] Reckelhoff JF. Gender differences in the regulation of blood pressure. Hypertension. 2001;37:1199–1208. doi: 10.1161/01.hyp.37.5.1199 11358929 10.1161/01.hyp.37.5.1199

[69] Visniauskas B Kilanowski-Doroh I Ogola BO McNally AB Horton AC Imulinde Sugi A Lindsey SH. Estrogen-mediated mechanisms in hypertension and other cardiovascular diseases. J Hum Hypertens. 2023;37:609–618. doi: 10.1038/s41371-022-00771-0 36319856 10.1038/s41371-022-00771-0 PMC10919324

