# Supplementary material for: Alcohol Intake and Risk of Hypertension: A Systematic Review and Dose-Response Meta-Analysis of Nonexperimental Cohort Studies
Source: Hypertension. 2024 Jun 12;81(8):1701–15. doi: 10.1161/HYPERTENSIONAHA.124.22703 (PMC11251509; doi:10.1161/HYPERTENSIONAHA.124.22703)
Supplement: Supplementary file 1 [file hyp-81-1701-s001.docx]

**Supplemental Material**

**Title:** Alcohol intake and risk of hypertension: a systematic review and dose-response meta-analysis of nonexperimental cohort studies

**Authors:** Marta Cecchini, MD^1^, Tommaso Filippini, MD, PhD^1,2^, Paul K. Whelton, MB, MD, MSc^3^, Inga Iamandii, MD^1^, Silvia Di Federico, MD^1^, Giuseppe Boriani, MD, PhD^4^, Marco Vinceti, MD, PhD^1,5^

**Affiliations:**

^1^CREAGEN - Environmental, Genetic and Nutritional Epidemiology Research Center, Section of Public Health, Department of Biomedical, Metabolic and Neural Sciences, University of Modena and Reggio Emilia, Modena, Italy;

^2^School of Public Health, University of California Berkeley, Berkeley, CA, USA;

^3^Department of Epidemiology, Tulane University School of Public Health and Tropical Medicine, New Orleans, LA, USA;

^4^Unit of Cardiology, Department of Biomedical, Metabolic and Neural Sciences, University of Modena and Reggio Emilia, Modena, Italy;

^5^Department of Epidemiology, Boston University School of Public Health, Boston, MA, USA.

**Available ORCIDs**: Tommaso Filippini: 0000-0003-2100-0344; Paul K Whelton: 0000-0002-2225-383X; Giuseppe Boriani: 0000-0002-9820-4815; Inga Iamandii: 0009-0008-1648-6128; Marco Vinceti: 0000-0002-0551-2473.

**Corresponding author:** Marco Vinceti. Department of Biomedical, Metabolic and Neural Sciences, University of Modena and Reggio Emilia, Via CAmpi 287, 41125 Modena, Italy; Tel. +390592055481. Fax: +390592055483. Mail: marco.vinceti@unimore.it

**Supplemental Methods**

This systematic review and meta-analysis were performed in accordance with the PRISMA (Preferred Reporting Items for Systematic Reviews and Meta-Analyses) guidelines ^1^. The study protocol was registered in the PROSPERO (no. CRD42022314389).

*Literature search and study selection*

We conducted a systematic literature search in PubMed and Embase, using the keywords: ‘’alcohol’’, “hypertension”, “blood pressure”, “stroke”, “humans”, “cohort”, “case-cohort” and ‘’English’’ or “Italian”, for non-experimental cohort studies published prior to February 19, 2024. Full literature search strategies are reported in Supplemental Table S1. Using the Population, Exposure, Comparison, Outcome and Study design (PECOS) approach ^2^, we included studies that reported the association between alcohol exposure and incidence of hypertension. We considered a study as eligible if it: 1) had employed a prospective cohort or cohort-nested case-control design, 2) had assessed both alcohol intake at baseline and incidence of hypertension during the follow-up; 3) included participants aged more than 18 years who were apparently healthy and without evidence of diseases or disorders such as cardiovascular disease or diabetes at the beginning of the study; 4) compared the risk of incident hypertension at two or more levels of alcohol exposure; 5) reported relative risk estimates as rate ratios (RRs), hazard ratios (HRs), or odds ratios (ORs), and provided a 95% confidence interval (CI); and 6) was written in English. We did not consider studies in which alcohol intake was expressed only as a continuous variable, thus providing risk estimates based on 1-unit increments in exposure based on a linear model because such studies are not suitable for the assessment of departure from linearity. We did not also consider studies based on binge alcohol drinking.

The titles/abstracts and full texts of studies identified in the original search were reviewed independently by three of the authors (MC, II, and SDF), with disagreements resolved by consensus or after review by two additional authors (TF and MV). The literature search was complemented by back and forward citation chasing based on the systematic scanning of the references of retrieved papers and of relevant systematic reviews and meta-analyses.

*Risk of bias assessment*

The internal validity of eligible studies was assessed independently by two authors (MC and TF) using the Risk of Bias for in Non-randomized Studies of Exposures (ROBINS-E) tool ^3^. Disagreements between the two assessors were resolved by consensus after review by a third author (MV). The following risk of bias domains were considered: (1) confounding, (2) selection of participants into the study, (3) exposure assessment (4) departure from intended exposure, (5) missing data, (6) outcome ascertainment, (7) selective reporting (Supplemental Table S2). The criteria used for the risk of bias assessment were tailored to address our specific research question and to accommodate the nature and methodology of the studies selected for our analysis, in accordance with the ROBINS-E guidance document ^3^. Thus, for each domain, studies were judged to be at low, moderate, or high risk of bias. Factors considered mandatory in order to judge a study as being at low risk of bias were age, adjustment for at least one factor among smoking habits, body mass index/waist circumference, and physical activity, selection of participants unrelated to alcohol exposure, exposure assessment performance using a validated food frequency questionnaire (FFQ) or interviewer-based questionnaire, availability of the exposure dose, missing data lower than 10%, external validation of the modality of outcome assessment, and clear reporting of the statistical methods. Studies were judged to be at moderate risk of bias if at least one of the previous criteria was missing for any category, and to be at high risk if either none of the factors were present, or the data were self-reported without an external validation. The overall results were tiered as follows: if at least one domain was found to identify a high risk of bias, the overall risk was considered high; if more than one domain was found to identify a moderate risk of bias, the overall risk was considered to be moderate; and if all domains were at low risk of bias, the overall risk was considered to be low.

**Supplemental Table S1**. Details of literature search on online databases.

| **Database** | **Search string** |
| --- | --- |
| PubMed | (ethanol[MH] OR alcohol[tiab] OR ethanol[tiab] OR "Alcoholic Beverages"[Mesh] OR "Alcoholic Beverage"[tiab]) AND ("Hypertension"[Mesh] OR Hypertension[tiab] OR 'blood pressure'[tiab] OR 'pressure, blood'[tiab] OR 'vascular pressure'[tiab] OR "Blood Pressure"[MeSH Terms] OR Stroke[MH] OR stroke[tiab]) AND humans[MH] AND ("Cohort Studies"[Mesh] OR cohort[tiab] OR case-cohort[tiab] OR prospective[tiab]) AND (English[Filter] OR italian[Filter]) |
| EMBASE | ('alcohol'/exp OR 'ethanol' OR 'alcoholic beverage'/exp OR 'alcoholic drink' OR 'liquor (alcohol)') AND ('hypertension'/exp OR 'blood pressure'/exp OR 'cerebrovascular accident'/exp) AND ('cohort analysis'/exp OR 'longitudinal study'/exp OR 'prospective study'/exp) AND [english]/lim AND [humans]/lim AND ([english]/lim OR [italian]/lim) |

**Supplemental Table S2**. Criteria adopted for risk of bias assessment using Risk of Bias for in Non-randomized Studies of Exposures (ROBINS-E) tool.

| **Domains** | **Criteria** |
| --- | --- |
| Bias due to confounding | Factors considered mandatory in order to judge a study at low risk of bias were: age and two factors among smoking habits, body mass index/waist circumference, and physical activity. Moderate risk if two are missing, high risk if three are missing or not reported. |
| Bias in selecting participants in the study | Selection of eligible participants not related to alcohol exposure for low risk. No criteria for moderate risk. |
| Bias in exposure classification | According to dietary intake assessment, studies were considered to be at low risk of bias if exposure assessment was performed using a validated food frequency questionnaire (FFQ) and interviewer-based questionnaire. Studies were considered to be at moderate risk of bias if exposure assessment was performed using a standardized questionnaire, or face-to-face surveys, or health modules. Studies were considered to be at high risk of bias if they used a self-administered questionnaire with no indication of a validation or criteria are not reported. |
| Bias in departure from intended exposure | Studies were categorized as having a low risk of bias if the exposure dose was reported, a moderate risk if the exposure levels were reported but not the doses in each category, and a high risk if alcohol levels were not reported. |
| Bias due to missing data | This required the definition of a reasonable cut-point for missing data. Studies with less than 10% missing data were considered to be at low risk and those with less than 20% to be at moderate risk. |
| Bias in outcome measurement | Possible bias was based on the modality of outcome assessment. A high risk was identified when the assessment was entirely based on self-report, without external validation, or no relevant information was reported. A moderate risk of bias was identified when the diagnosis was reported using ICD classification but there was no indication of the cut-points used for diagnosis of hypertension. |
| Bias in selection of reported results | This assessment was based on the evidence that the study results had not been selected. Clear reporting of statistical methods was required for designation of a low risk of bias. When no criteria were reported, the study was identified as having a moderate risk of bias. |
| Overall risk of bias | If at least one domain was found to identify a high risk of bias, the overall risk was considered to be high. If more than one domain was found to reflect a moderate risk of bias, the overall risk was considered to be moderate. If all the domains were identified as being at low risk of bias, the overall risk was considered to be low. |

**Supplemental Table S3**. Results of risk of bias (RoB) assessment.

| **Studies** | **Bias due to confounding** | **Bias in selecting participants in the study** | **Bias in exposure classification** | **Bias in departure from intended exposure** | **Bias due to missing data** | **Bias in outcome measurement** | **Bias in selection of reported results** | **Study-level RoB Judgment** |
| --- | --- | --- | --- | --- | --- | --- | --- | --- |
| Bai  2017^4^ | Low | Low | Moderate | Low | Low | Moderate | Low | Moderate |
| Banda  2010^5^ | Low | Low | Moderate | Low | Low | Low | Low | Moderate |
| Curtis 1997^6^ | Moderate | Low | Low | Low | Low | Low | Low | Moderate |
| Fuchs 2001^7^ | Low | Low | Low | Low | Moderate | Low | Low | Moderate |
| Halanych  2010^8^ | Low | Low | Moderate | Low | Low | Low | Low | Moderate |
| Im 2023^9^ | Moderate | Low | Moderate | Low | Moderate | Moderate | Low | Moderate |
| Jung  2020^10^ | Low | Low | Low | Low | Low | Low | Low | Low |
| Lee  2005^11^ | Low | Low | High | Low | Moderate | Low | Low | High |
| Lui  2020^12^ | High | Low | Moderate | Low | Low | High | Low | High |
| Nagao  2021^13^ | Low | Low | Moderate | Low | Low | Low | Low | Moderate |
| Nakanishi  2001^14^ | Low | Low | Low | Low | Low | Low | Low | Low |
| Niskanen  2004^15^ | Low | Low | High | Low | Low | Low | Low | High |
| Núñez-Córdoba  2009^16^ | Low | Low | Low | Low | Moderate | Low | Low | Moderate |
| Ohmori  2002^17^ | Moderate | Low | Moderate | Low | Low | Low | Low | Moderate |
| Okubo  2014^18^ | Low | Low | Low | Low | Low | Low | Low | Low |
| Peng  2013^19^ | Low | Low | Moderate | Low | Low | Low | Low | Moderate |
| Qiu  2022^20^ | Low | Low | Moderate | Low | Low | Moderate | Low | Moderate |
| Saremi  2004^21^ | Moderate | Low | Moderate | Low | Low | Low | Low | Moderate |
| Sesso  2008^22^ | Low | Low | Low | Low | Low | Low | Low | Low |
| Thadhani  2002^23^ | Low | Low | Low | Low | Moderate | Low | Low | Moderate |
| Wang  2022^24^ | Low | Low | Moderate | Low | Low | Low | Low | Moderate |
| Witteman  1990^25^ | Moderate | Low | Low | Low | Moderate | Low | Low | Moderate |
| Yoo  2019^26^ | Low | Low | Low | Low | Low | Low | Low | Low |

**Supplemental Figure S1.** Forest plot for the association between alcohol consumption and incident hypertension in 22 cohort studies. The area of each gray square is proportional to the inverse of the variance of the estimated log RR resulting that the size of the square is positively associated with sample size of the study population, whereas horizontal line represents the 95% prediction interval intervals (CIs). Vertical axis of the gray diamonds represents the point estimate of the overall RR and the vertical axis its 95% CI. The solid vertical line represents RR=1. CI, confidence interval; M, men; MB, men-Black population; MH, men-Hispanic population; MW, men-White population; RR, risk ratio; W, women; WB, women-Black population; WH, women-Hispanic population; WW, women-White population.


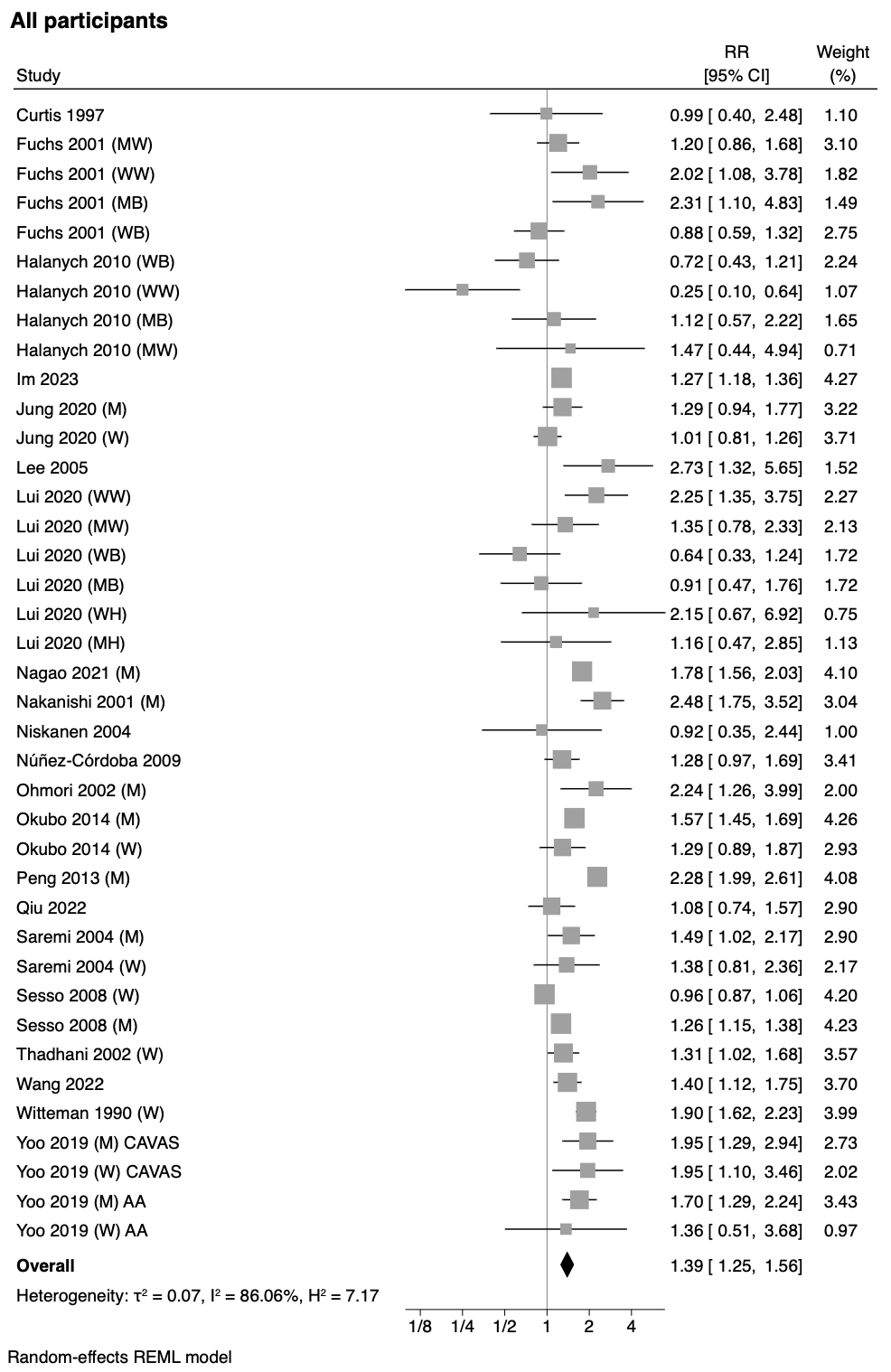


**Supplemental Figure S2.** Forest plot for the association between alcohol consumption and incident hypertension in men (18 studies). The area of each gray square is proportional to the inverse of the variance of the estimated log RR resulting that the size of the square is positively associated with sample size of the study population, whereas horizontal line represents the 95% prediction interval intervals (CIs). Vertical axis of the gray diamonds represents the point estimate of the overall RR and the vertical axis its 95% CI. The solid vertical line represents RR=1. CI, confidence interval; M, men; MB, men-Black population; MH, men-Hispanic population; MW, men-White population; RR, risk ratio.


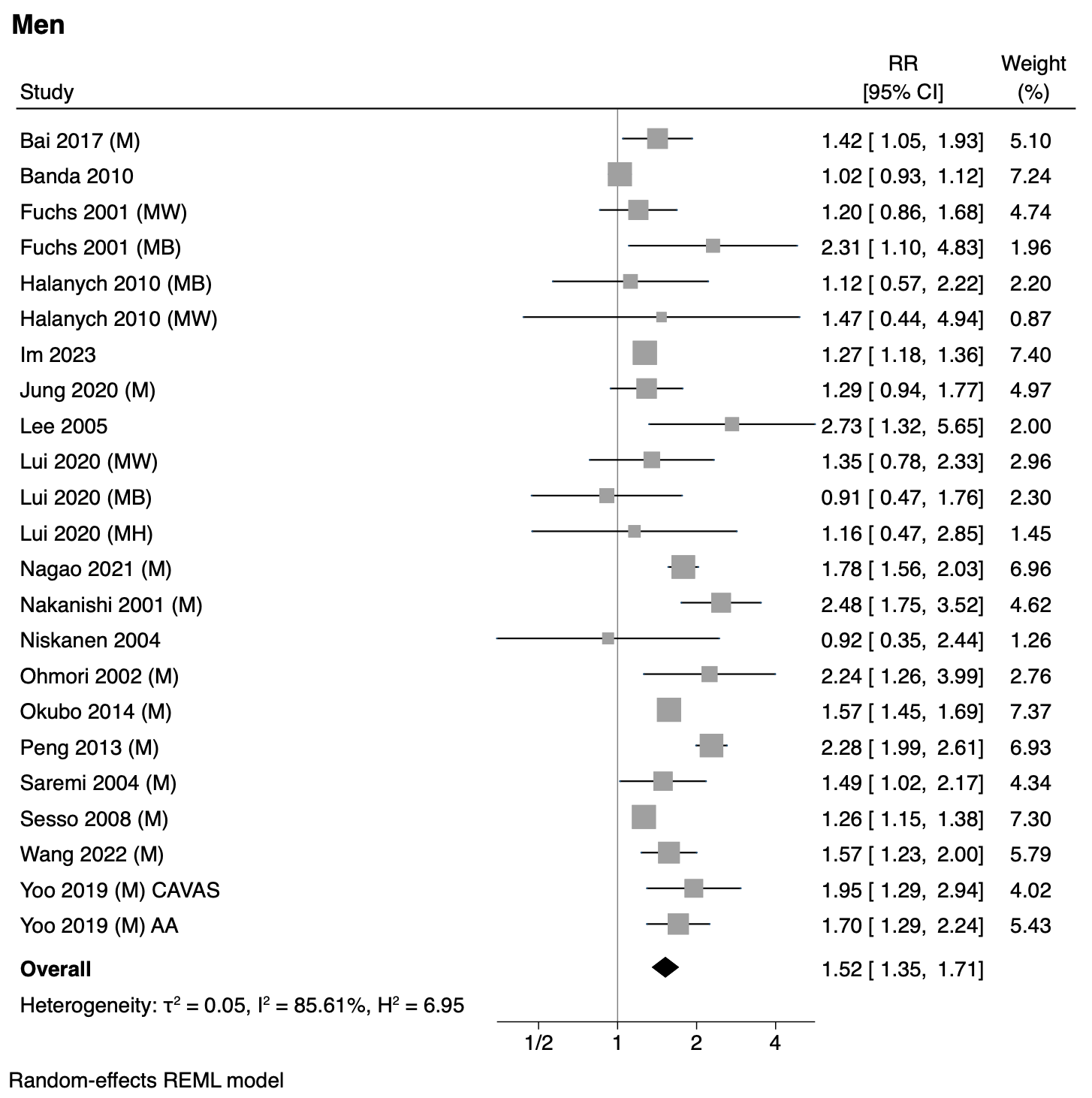


**Supplemental Figure S3.** Forest plot for the association between alcohol consumption and incident hypertension in women (12 studies). The area of each gray square is proportional to the inverse of the variance of the estimated log RR resulting that the size of the square is positively associated with sample size of the study population, whereas horizontal line represents the 95% prediction interval intervals (CIs). Vertical axis of the gray diamonds represents the point estimate of the overall RR and the vertical axis its 95% CI. The solid vertical line represents RR=1. CI, confidence interval; RR, risk ratio; W, women; WB, women-Black population; WH, women-Hispanic population; WW, women-White population.


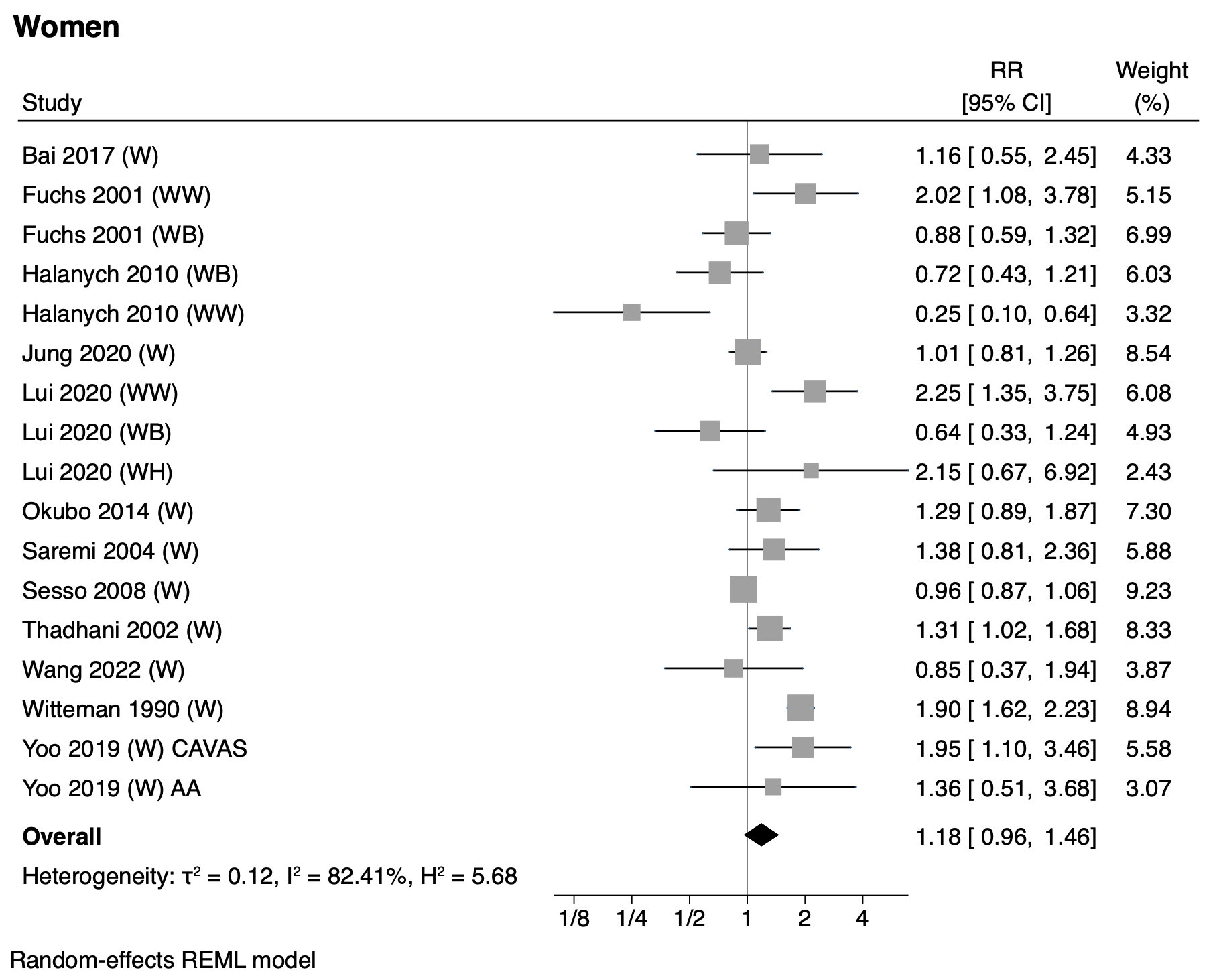


**Supplemental Figure S4** Dose-response meta-analysis of the risk of hypertension according to alcohol consumption after removing the 3 studies at high risk of bias. Analysis presented in (A) all study participants (n=19 studies), (B) men (n=15 studies) and (C) women (n=11 studies). Overall spline curve (black solid line) with 95% confidence limits (grey area).


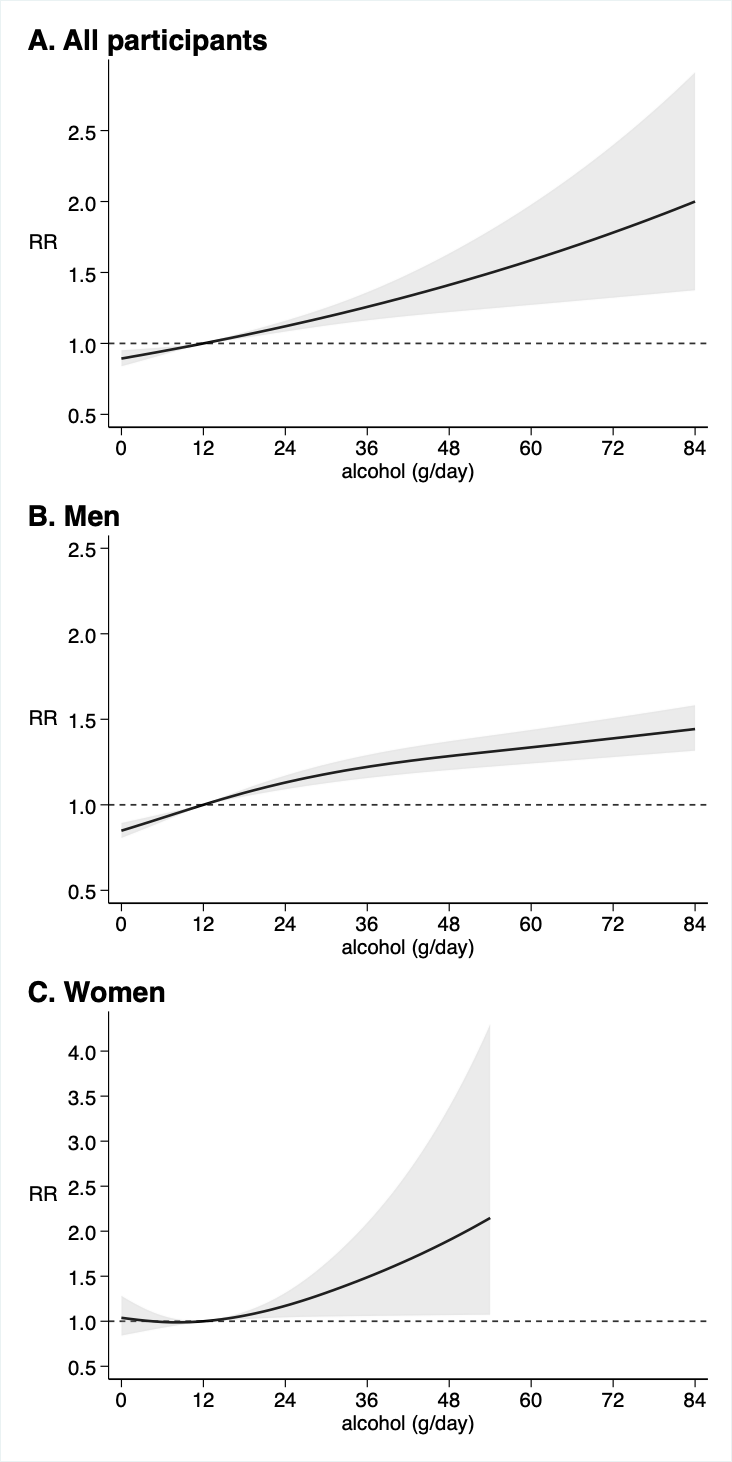


**Supplemental Figure S5** Dose-response meta-analysis of the risk of hypertension according to alcohol consumption after removing the 3 studies with different cutoffs for definition of hypertension or not reporting them. Analysis presented in (A) all study participants (n=19 studies), (B) men (n=16 studies) and (C) women (n=10 studies). Overall spline curve (black solid line) with 95% confidence limits (grey area).

**
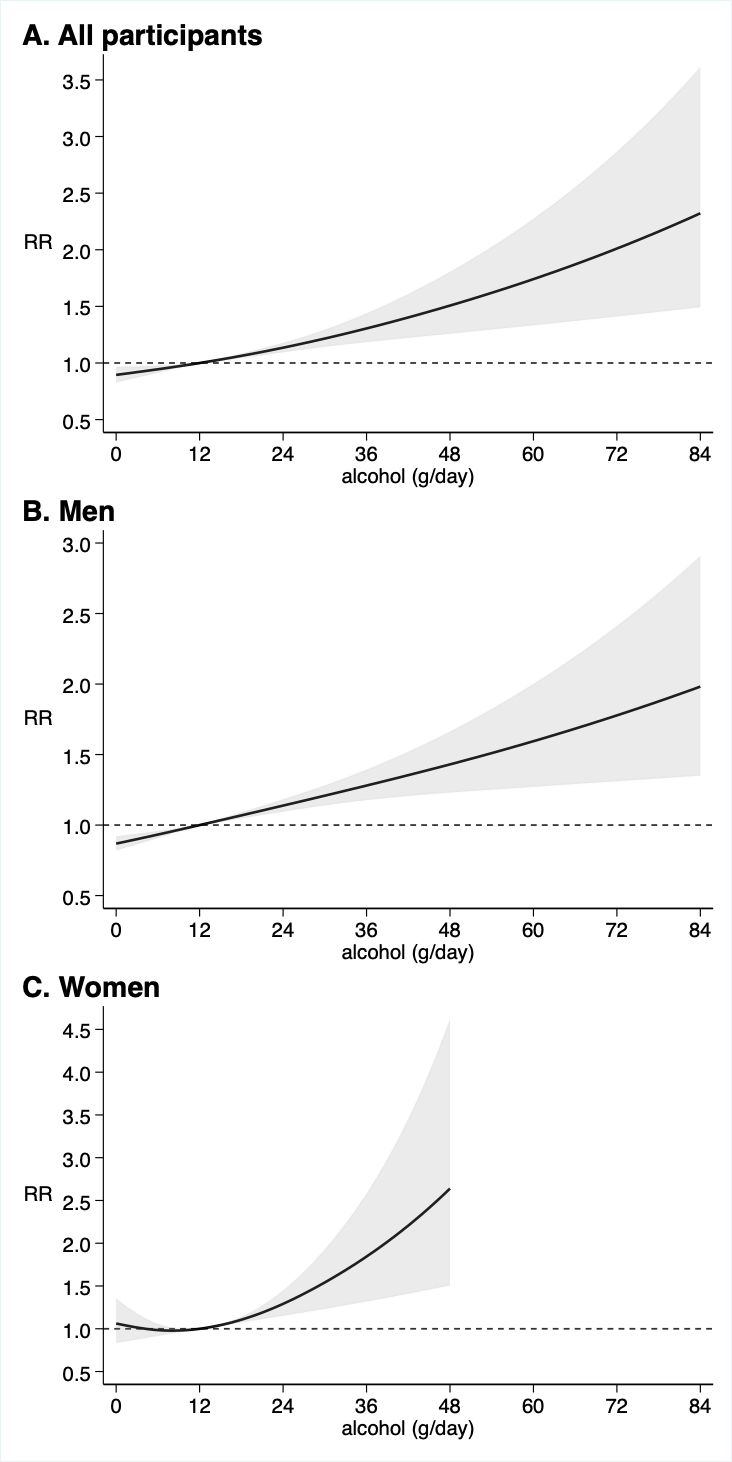
**

**Supplemental Figure S6** Dose-response meta-analysis of the risk of hypertension according to alcohol consumption after removing the studies not adjusting for smoking. Analysis presented in (A) all study participants (n=16 studies), (B) men (n=14 studies) and (C) women (n=8 studies). Overall spline curve (black solid line) with 95% confidence limits (grey area).

**
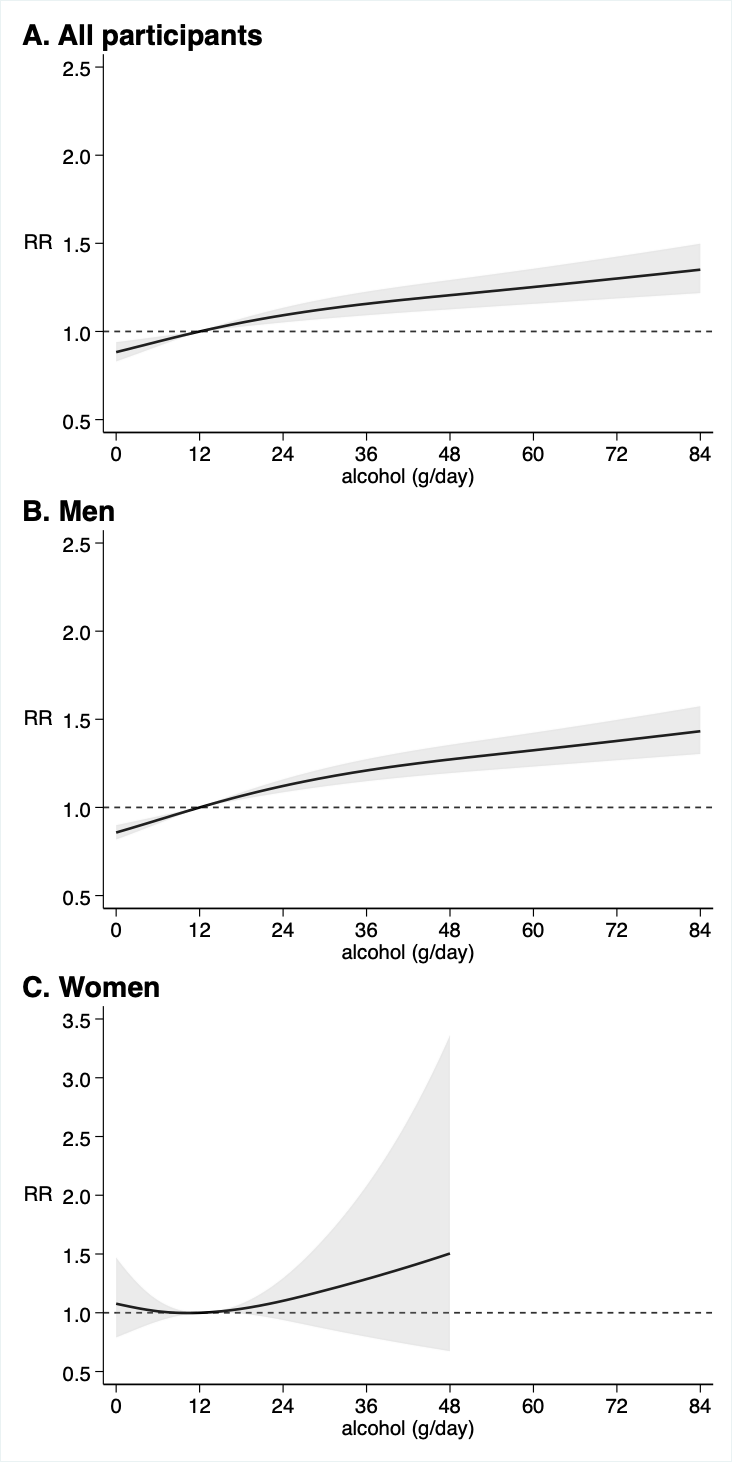
**

**Supplemental Figure S7** Dose-response meta-analysis of the risk of hypertension according to alcohol consumption divided by duration of follow-up. Analysis presented stratified in (A) <20 years (19 studies), and (B) ≥20 years (4 studies). Overall spline curve (black solid line) with 95% confidence limits (grey area).

**
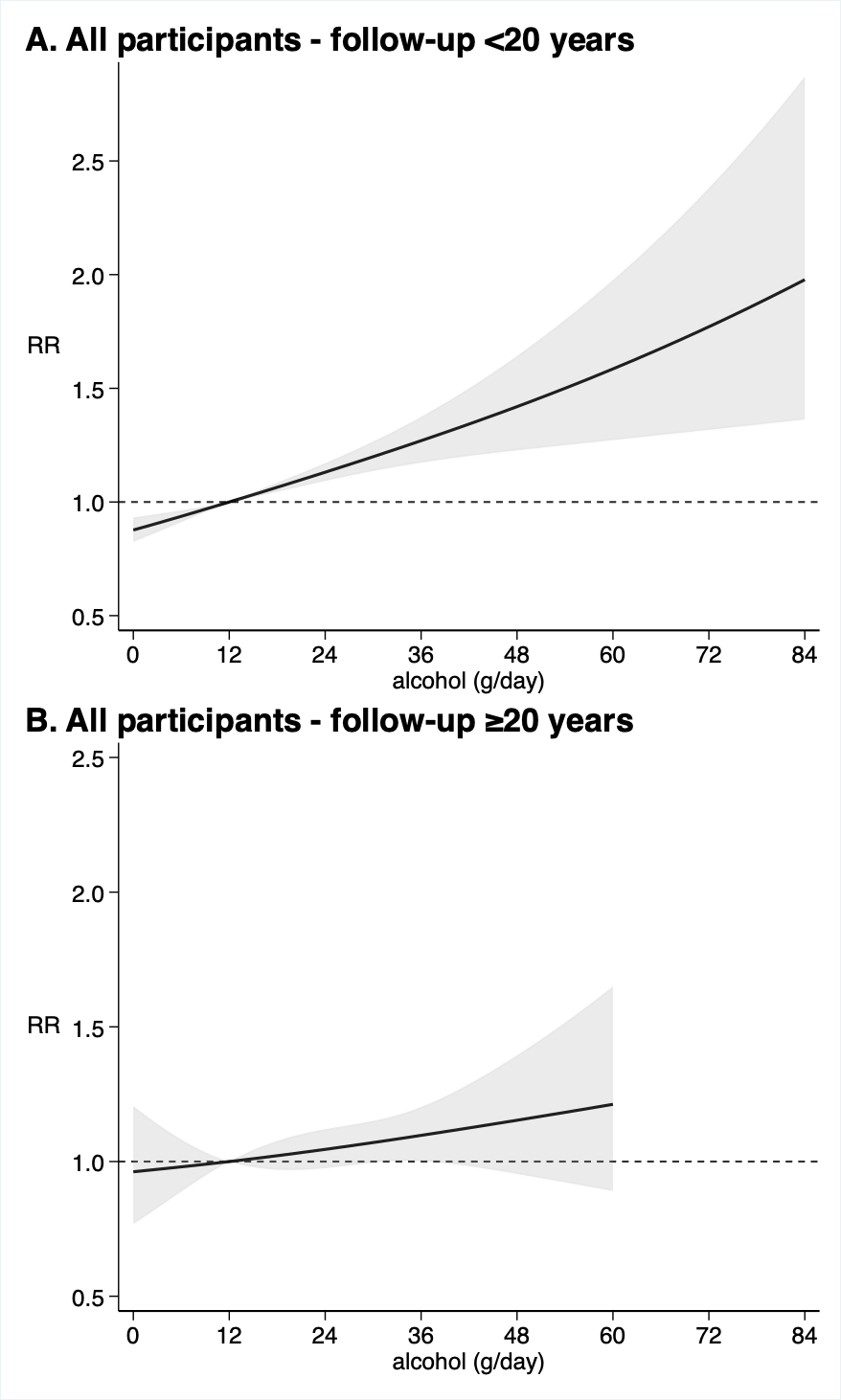
**

**Supplemental Figure S8.** Sensitivity analysis of study-specific trends of the d
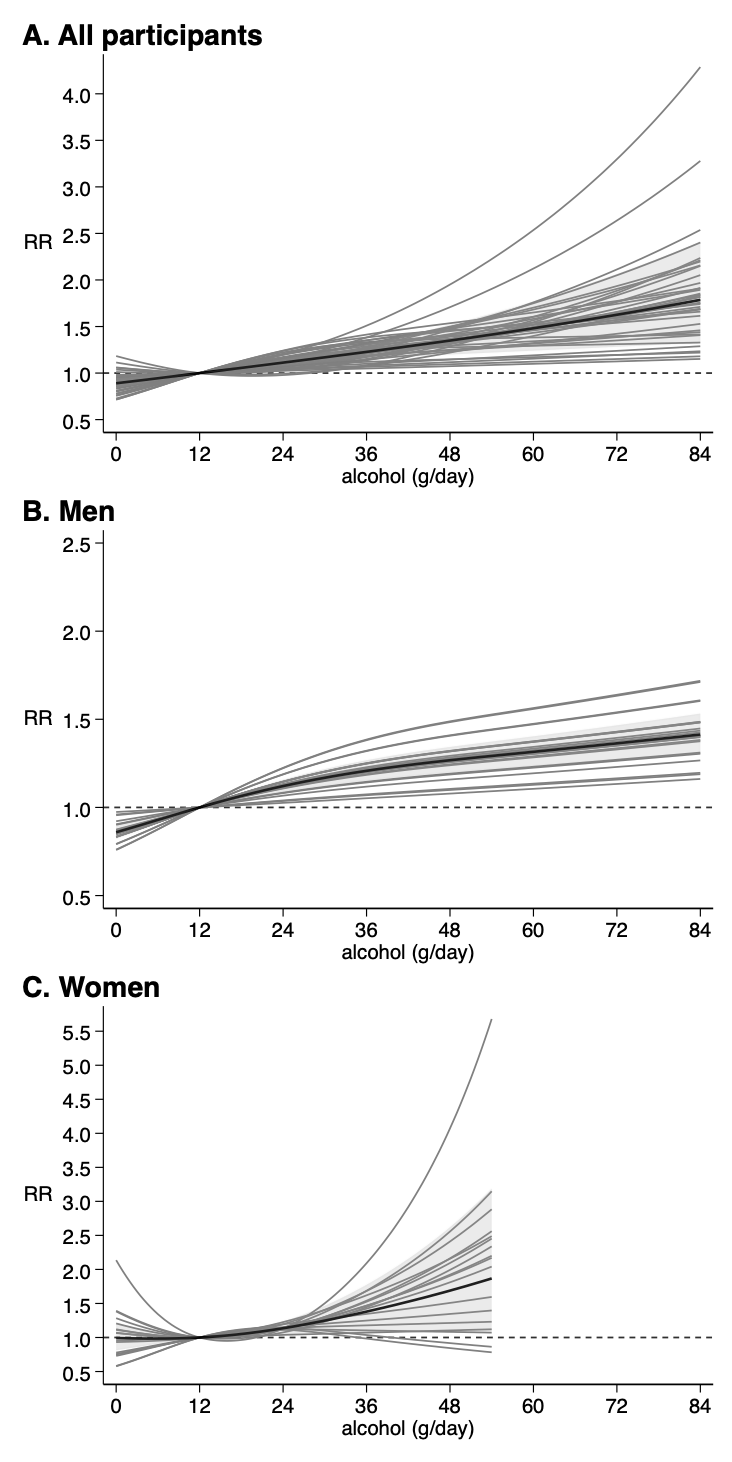
ose-response meta-analysis between alcohol intake and risk of hypertension according to alcohol consumption. Analyses presented in (A) all study participants (n=22 studies), in (B) men (n=18) and in (C) women (n=12). Overall spline curve (black solid line) with 95% confidence limits (grey area) and the study-specific trends showing the influence of variation across studies (grey solid lines). RR: risk ratio.


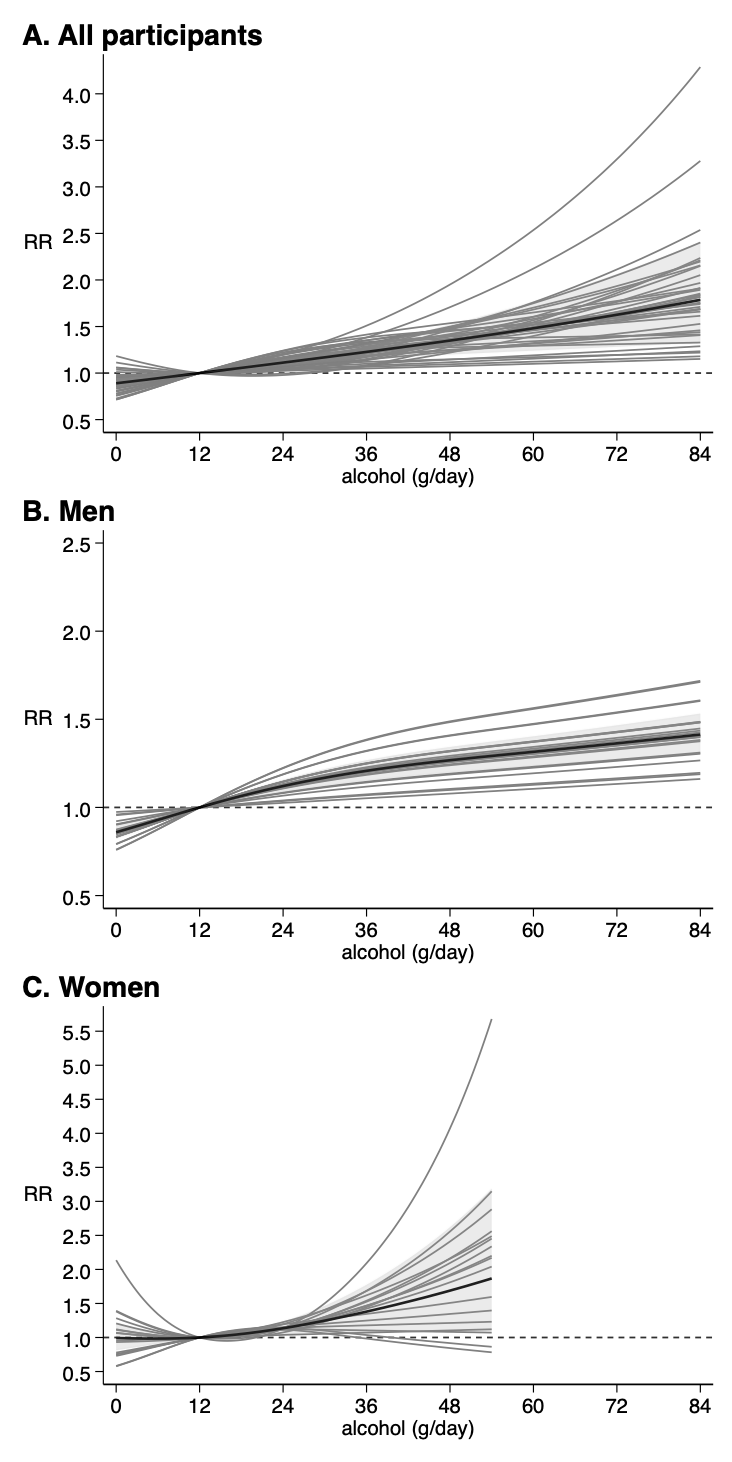


**Supplemental Figure S9.** Funnel plot for publication bias and small-study effects. Analysis presented in (A) all study participants, (B) men and (C) women.


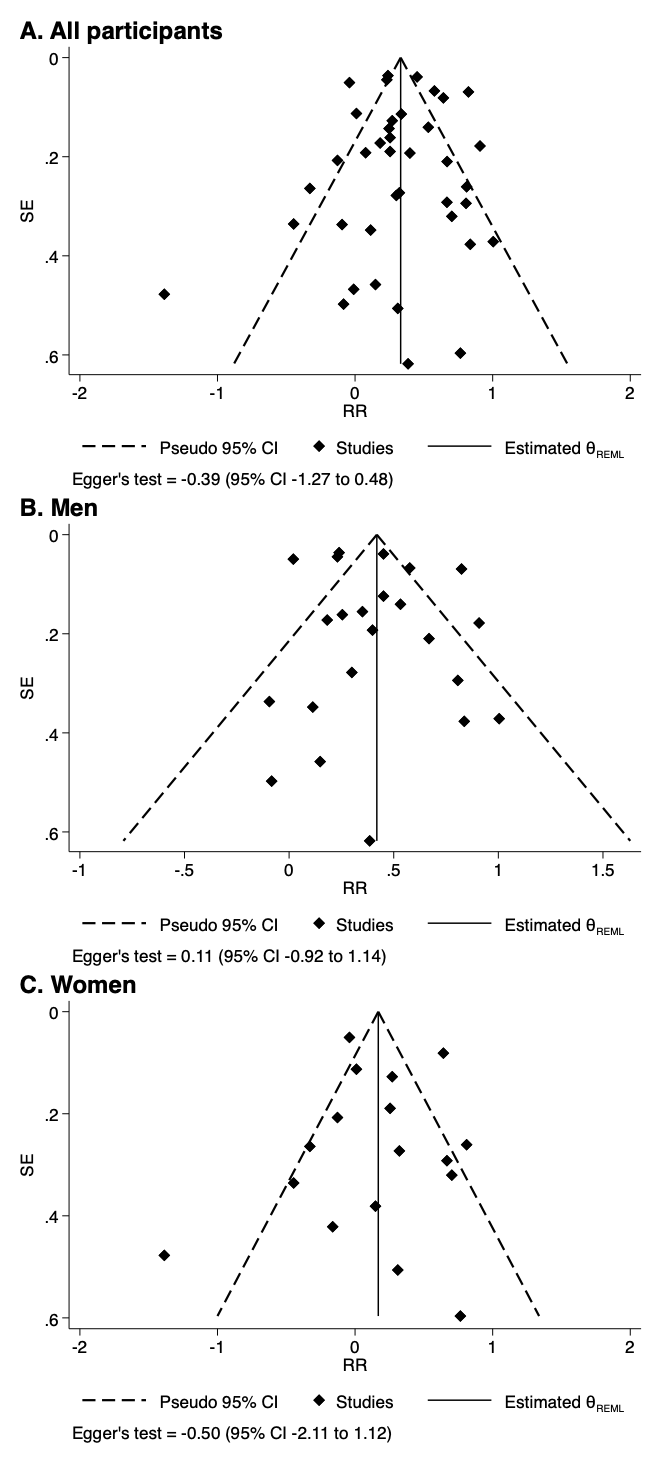


**Supplemental References**

1. Page MJ, McKenzie JE, Bossuyt PM, Boutron I, Hoffmann TC, Mulrow CD, Shamseer L, Tetzlaff JM, Akl EA, Brennan SE et al. The PRISMA 2020 statement: an updated guideline for reporting systematic reviews. *BMJ*. 2021;372:n71. doi:10.1136/bmj.n71.

2. Morgan RL, Whaley P, Thayer KA, Schunemann HJ. Identifying the PECO: a framework for formulating good questions to explore the association of environmental and other exposures with health outcomes. *Environ Int*. 2018;121:1027-1031. doi:10.1016/j.envint.2018.07.015.

3. Morgan RL, Thayer KA, Santesso N, Holloway AC, Blain R, Eftim SE, Goldstone AE, Ross P, Ansari M, Akl EA et al. A risk of bias instrument for non-randomized studies of exposures: a users' guide to its application in the context of GRADE. *Environ Int*. 2019;122:168-184. doi:10.1016/j.envint.2018.11.004.

4. Bai G, Zhang J, Zhao C, Wang Y, Qi Y, Zhang B. Adherence to a healthy lifestyle and a DASH-style diet and risk of hypertension in Chinese individuals. *Hypertens Res*. 2017;40:196-202. doi:10.1038/hr.2016.119.

5. Banda JA, Clouston K, Sui X, Hooker SP, Lee CD, Blair SN. Protective health factors and incident hypertension in men. *Am J Hypertens*. 2010;23:599-605. doi:10.1038/ajh.2010.26.

6. Curtis AB, James SA, Strogatz DS, Raghunathan TE, Harlow S. Alcohol consumption and changes in blood pressure among African Americans. The Pitt County Study. *Am J Epidemiol*. 1997;146:727-733. doi:10.1093/oxfordjournals.aje.a009348.

7. Fuchs FD, Chambless LE, Whelton PK, Nieto FJ, Heiss G. Alcohol consumption and the incidence of hypertension: the Atherosclerosis Risk in Communities Study. *Hypertension*. 2001;37:1242-1250. doi:10.1161/01.hyp.37.5.1242.

8. Halanych JH, Safford MM, Kertesz SG, Pletcher MJ, Kim YI, Person SD, Lewis CE, Kiefe CI. Alcohol consumption in young adults and incident hypertension: 20-year follow-up from the Coronary Artery Risk Development in Young Adults Study. *Am J Epidemiol*. 2010;171:532-539. doi:10.1093/aje/kwp417.

9. Im PK, Wright N, Yang L, Chan KH, Chen Y, Guo Y, Du H, Yang X, Avery D, Wang S et al. Alcohol consumption and risks of more than 200 diseases in Chinese men. *Nat Med*. 2023;29:1476-1486. doi:10.1038/s41591-023-02383-8.

10. Jung S, Kim MK, Shin J, Lee N, Woo HW, Choi BY, Shin MH, Shin DH, Lee YH. Positive association of alcohol consumption with incidence of hypertension in adults aged 40 years and over: use of repeated alcohol consumption measurements. *Clin Nutr*. 2020;39:3125-3131. doi:10.1016/j.clnu.2020.01.020.

11. Lee SH, Kim YS, Sunwoo S, Huh BY. A retrospective cohort study on obesity and hypertension risk among Korean adults. *J Korean Med Sci*. 2005;20:188-195. doi:10.3346/jkms.2005.20.2.188.

12. Lui CK, Kerr WC, Li L, Mulia N, Ye Y, Williams E, Greenfield TK, Lown EA. Lifecourse drinking patterns, hypertension, and heart problems among U.S. adults. *Am J Prev Med*. 2020;58:386-395. doi:10.1016/j.amepre.2019.10.018.

13. Nagao T, Nogawa K, Sakata K, Morimoto H, Morita K, Watanabe Y, Suwazono Y. Effects of alcohol consumption and smoking on the onset of hypertension in a long-term longitudinal study in a male workers' cohort. *Int J Environ Res Public Health*. 2021;18:11781. doi:10.3390/ijerph182211781.

14. Nakanishi N, Yoshida H, Nakamura K, Suzuki K, Tatara K. Alcohol consumption and risk for hypertension in middle-aged Japanese men. *J Hypertens*. 2001;19:851-855. doi:10.1097/00004872-200105000-00003.

15. Niskanen L, Laaksonen DE, Nyyssonen K, Punnonen K, Valkonen VP, Fuentes R, Tuomainen TP, Salonen R, Salonen JT. Inflammation, abdominal obesity, and smoking as predictors of hypertension. *Hypertension*. 2004;44:859-865. doi:10.1161/01.HYP.0000146691.51307.84.

16. Nunez-Cordoba JM, Martinez-Gonzalez MA, Bes-Rastrollo M, Toledo E, Beunza JJ, Alonso A. Alcohol consumption and the incidence of hypertension in a Mediterranean cohort: the SUN study. *Rev Esp Cardiol*. 2009;62:633-641. doi:10.1016/s1885-5857(09)72227-3.

17. Ohmori S, Kiyohara Y, Kato I, Kubo M, Tanizaki Y, Iwamoto H, Nakayama K, Abe I, Fujishima M. Alcohol intake and future incidence of hypertension in a general Japanese population: the Hisayama study. *Alcohol Clin Exp Res*. 2002;26:1010-1016. doi:10.1097/01.ALC.0000021147.31338.C2.

18. Okubo Y, Sairenchi T, Irie F, Yamagishi K, Iso H, Watanabe H, Muto T, Tanaka K, Ota H. Association of alcohol consumption with incident hypertension among middle-aged and older Japanese population: the Ibarakai Prefectural Health Study (IPHS). *Hypertension*. 2014;63:41-47. doi:10.1161/HYPERTENSIONAHA.113.01585.

19. Peng M, Wu S, Jiang X, Jin C, Zhang W, Kailuan Cardiovascular Survey G. Long-term alcohol consumption is an independent risk factor of hypertension development in northern China: evidence from Kailuan study. *J Hypertens*. 2013;31:2342-2347. doi:10.1097/HJH.0b013e3283653999.

20. Qiu W, Cai A, Li L, Feng Y. Longitudinal trajectories of alcohol consumption with all-cause mortality, hypertension, and blood pressure change: results from CHNS Cohort, 1993-2015. *Nutrients*. 2022;14:5073. doi:10.3390/nu14235073.

21. Saremi A, Hanson RL, Tulloch-Reid M, Williams DE, Knowler WC. Alcohol consumption predicts hypertension but not diabetes. *J Stud Alcohol*. 2004;65:184-190. doi:10.15288/jsa.2004.65.184.

22. Sesso HD, Cook NR, Buring JE, Manson JE, Gaziano JM. Alcohol consumption and the risk of hypertension in women and men. *Hypertension*. 2008;51:1080-1087. doi:10.1161/HYPERTENSIONAHA.107.104968.

23. Thadhani R, Camargo CA, Jr., Stampfer MJ, Curhan GC, Willett WC, Rimm EB. Prospective study of moderate alcohol consumption and risk of hypertension in young women. *Arch Intern Med*. 2002;162:569-574. doi:10.1001/archinte.162.5.569.

24. Wang Y, Yao Y, Chen Y, Zhou J, Wu Y, Fu C, Wang N, Liu T, Xu K. Association between drinking patterns and incident hypertension in Southwest China. *Int J Environ Res Public Health*. 2022;19:3801. doi:10.3390/ijerph19073801.

25. Witteman JC, Willett WC, Stampfer MJ, Colditz GA, Kok FJ, Sacks FM, Speizer FE, Rosner B, Hennekens CH. Relation of moderate alcohol consumption and risk of systemic hypertension in women. *Am J Cardiol*. 1990;65:633-637. doi:10.1016/0002-9149(90)91043-6.

26. Yoo MG, Park KJ, Kim HJ, Jang HB, Lee HJ, Park SI. Association between alcohol intake and incident hypertension in the Korean population. *Alcohol*. 2019;77:19-25. doi:10.1016/j.alcohol.2018.09.002.
